# Hibiscus Mutabilis‐Inspired Upcycled TPEE Films with Orthogonal Wavelength‐Controlled Spiropyrans for Dynamic Anticounterfeiting and Photoswitchable Conductivity

**DOI:** 10.1002/smll.202503829

**Published:** 2025-07-22

**Authors:** Yi‐Fan Chen, Lin‐Ruei Lee, Ming‐Hsuan Chang, Huan‐Wei Lin, Yu‐Chun Liu, Chun‐Ting Chang, Kai‐Chuan Kuo, Chun‐Chi Chang, Che‐Tseng Lin, Jiun‐Tai Chen

**Affiliations:** ^1^ Department of Applied Chemistry National Yang Ming Chiao Tung University Hsinchu 300093 Taiwan; ^2^ Department of Performance Materials Synthesis & Application Division of Polymer Research Material and Chemical Research Laboratories Industrial Technology Research Institute Hsinchu 300044 Taiwan; ^3^ Center for Emergent Functional Matter Science National Yang Ming Chiao Tung University Hsinchu 300093 Taiwan

**Keywords:** anticounterfeiting, conductivity, photoswitchable, spiropyran, upcycling polymer

## Abstract

Plastics are integral to modern life but contribute significantly to environmental pollution due to their durability and low recycling rates. Poly(ethylene terephthalate) (PET) is particularly problematic, representing a substantial portion of global plastic waste. Addressing this issue, this work presents a sustainable approach to upcycle PET into thermoplastic polyester elastomer (TPEE) films, integrated with orthogonal wavelength‐controlled spiropyran derivatives, SP‐COOH and MC‐SO_3_. These upcycled TPEE films exhibit dynamic anticounterfeiting and photoswitchable conductivity properties. The spiropyran derivatives demonstrate reversible structural changes and color variations under different light conditions, enhancing their mechanical properties and solvent resistance. Inspired by the color changing of *Hibiscus mutabilis*, this work also demonstrates the artificial light‐induced color on the petals of moth orchids. The incorporation of conductive poly(3,4‐ethylenedioxythiophene) polystyrene sulfonate (PEDOT:PSS) further enables photoswitchable conductivity, offering a dual‐layered security feature. This work not only advances polymer upcycling but also introduces innovative applications in anticounterfeiting and wearable technology, aligning with global sustainability goals and demonstrating the potential for high‐value product development from waste materials.

## Graphical abstract

PET is upcycled into thermoplastic polyester elastomer (TPEE) films, integrated with orthogonal wavelength‐controlled spiropyran derivatives. These upcycled TPEE films exhibit dynamic anticounterfeiting and photoswitchable conductivity properties under different light conditions. Inspired by the color changing of *Hibiscus mutabilis*, the artificial light‐induced color on the petals of moth orchids is also demonstrated.

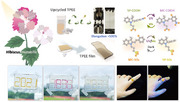

## 1 Introduction

Plastics are ubiquitous in daily life because of their low manufacturing costs, lightweight nature, and high stability.^[^ ^1^ ^]^ According to the “Plastics‐the Facts” report, global plastics production surged from 225 to 400 million metric tonnes between 2004 and 2022.^[^ ^2^ ^]^ The durability of plastics, however, means that they persist in the environment for hundreds of years, contributing significantly to pollution.^[^ ^3^, ^4^ ^]^ Among the vast quantities produced, less than 0.1% of plastics are derived from chemically recycled polymers.^[^ ^2^ ^]^ Additionally, toxic contaminants from plastic waste severely threaten humans, animals, plants, and marine life.^[^ ^5^, ^6^ ^]^ For various plastic products, poly(ethylene terephthalate) (PET) stands out as one of the most widely produced, accounting for approximately month12% of total solid waste, particularly for food packaging and clothing.^[^ ^7^ ^]^ Consequently, managing PET waste has become a critical environmental challenge that requires urgent attention. To address this issue, several solutions have been proposed to enhance PET recycling techniques, such as catalysts,^[^ ^8^ ^]^ enzymes,^[^ ^9^ ^]^ and methanolysis.^[^ ^10^ ^]^

In addition to plastic recycling, the concept of “upcycling” has emerged, referring to processes that convert by‐products, unwanted materials, or waste into new, higher‐value products.^[^ ^1^, ^11^ ^]^ This approach aims to add value to waste materials, further contributing to sustainable waste management practices. For PET wastes, research has also bloomed to repropose the materials, demonstrating the applications in Li‐ion batteries,^[^ ^12^ ^]^ ammonia electrosynthesis,^[^ ^13^ ^]^ hydrogen fuel,^[^ ^14^, ^15^ ^]^ and glycolic acid formation.^[^ ^16^ ^]^ These studies, however, are mainly based on the category “from polymer to fine chemicals” and their applications related to the product.^[^ ^17^ ^]^ For recycled PET, several studies have reported relatively low mechanical properties due to the greater degradation of PET chains during recycling compared to other thermoplastics, limiting its further applications.^[^ ^18^, ^19^ ^]^ Hence, the upcycled materials “from PET polymer to polymer” with better mechanical properties, chemical/solvent corrosion resistance, and some further applications still need to be discovered.

From software to currency, counterfeit goods in the market result in both economic loss and copyright infringement; moreover, potential health risks can highly arise in industries such as food packaging and nongenuine medicines.^[^ ^20^, ^21^ ^]^ According to reports from the Organization for Economic Co‐operation and Development (OECD),^[^ ^22^ ^]^ imports of counterfeit and pirated goods reached USD 464 billion in 2019, which is up to 2.5% of global trade. To solve this problem, the rapid development of anticounterfeiting materials, including self‐assembling,^[^ ^23^, ^24^ ^]^ nanoparticles,^[^ ^25^, ^26^, ^27^ ^]^ organic or inorganic materials,^[^ ^28^, ^29^, ^30^, ^31^, ^32^ ^]^ and carbon dots,^[^ ^33^ ^]^ have been explored for developing next‐generation technologies. Among these materials, photoluminescence molecules stand out because of their ease of use, high throughput, straightforward design, and adjustable optical properties across various dimensions, making them widely adopted.^[^ ^20^, ^34^, ^35^ ^]^ With different designs, such as wavelengths, polarized light, deformation, and patterns, the information presented by photoluminescence molecules can be easily and independently read by the naked eye.^[^ ^36^, ^37^, ^38^, ^39^ ^]^ Unlike other molecules,^[^ ^40^, ^41^ ^]^ spiropyran shows vivid color changes with photoisomerization under UV and visible light and switches properties with exposure to acid or base vapors.^[^ ^42^ ^]^ Devices utilizing spiropyran, however, typically involve complex reactions, polymerizations, or assemblies, posing challenges to industrial scalability. Thus, there is increasing demand for simplified fabrication processes for spiropyran‐based anticounterfeiting materials, especially using recycled or upcycled materials to meet sustainability goals.

To address these issues, herein, we explore a feasible and versatile approach to prepare upcycled anticounterfeiting materials by coating orthogonal wavelength‐controlled spiropyrans (SP) on thermoplastic polyester elastomer (TPEE) films. Two spiropyran derivatives, SP‐COOH and SP‐SO_3_, are chosen to possess different structural changes upon light irradiation. With different wavelengths of 365 nm, 555 nm, and darkness, the structural variation between SP‐COOH and SP‐SO_3_ can be reversibly controlled in solution states. Upcycled TPEE materials are fabricated from recycled PET scraps and further applied by spin‐coating, enhancing better mechanical properties, including higher stretchability, solvent corrosive resistance, and durability. Moreover, we demonstrate the anticounterfeiting effect based on solid‐state regulation of the spiropyran dyes incorporated with thickeners coated on the TPEE films with designed information patterns. While irradiated with different lights, various patterns can be identified accordingly and reversibly; meanwhile, the photoswitchable properties can be demonstrated under tension, providing potential applications on wearable devices. Inspired by the color changing of *Hibiscus mutabilis*, we also demonstrate the artificial light‐induced color on the petals of moth orchids. Furthermore, applying conductive poly(3,4‐ethylenedioxythiophene) polystyrene sulfonate (PEDOT:PSS) into the photoswitchable inks can also induce resistance changes during cycles of UV and visible lights, which can be a second lock for the information encryption. To the best of our knowledge, this work is the first study to harness the innovative potential of upcycled polymers with photoswitchable, orthogonal wavelength‐controlled spiropyrans, making it an excellent candidate in the fields of polymer upcycling and information encryption.

## 2 Results and Discussion

**Figure 1:**
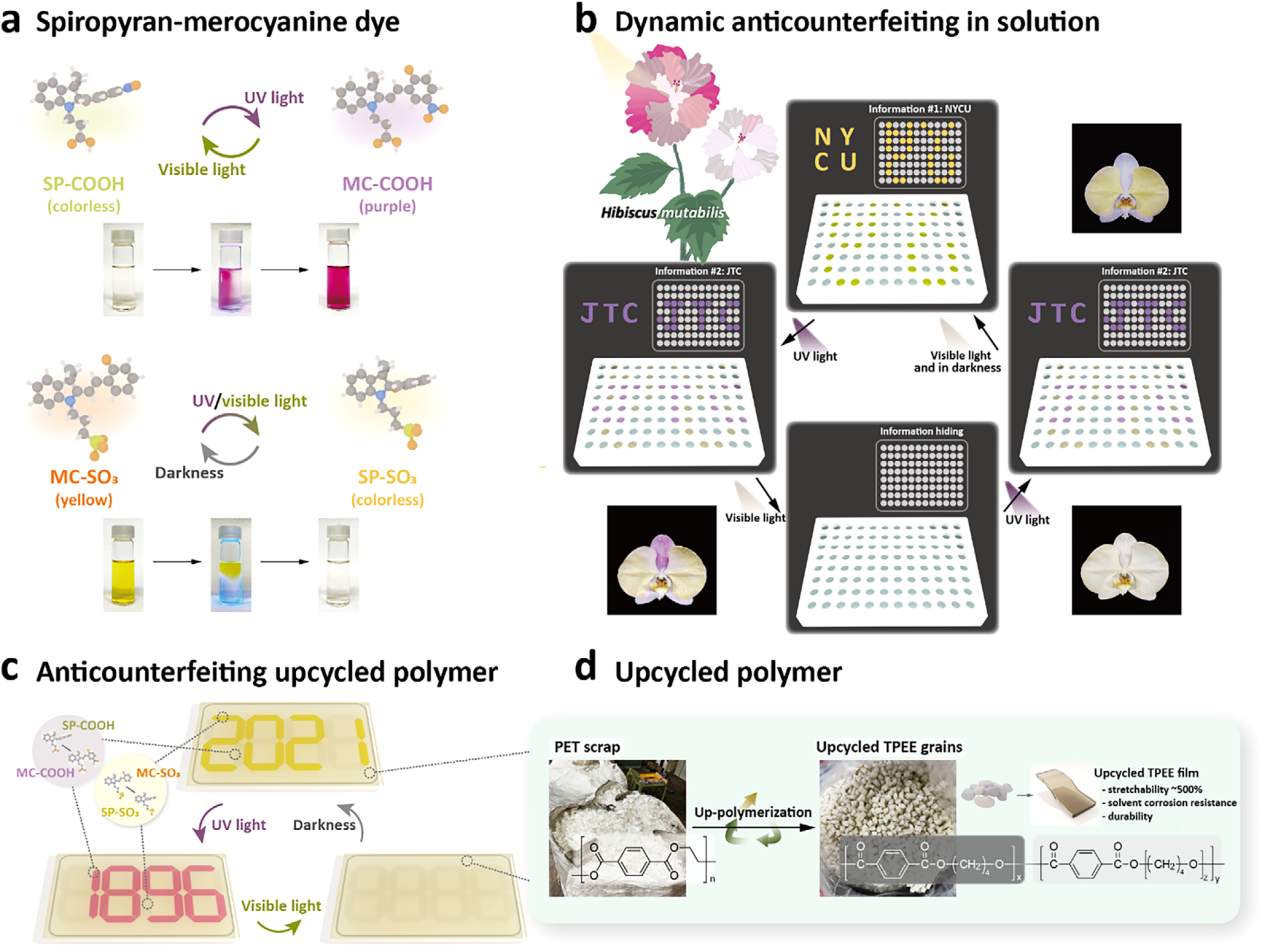
Conceptual design and mechanism illustration of the upcycled TPEE polymer as anti‐counterfeiting materials. a) Schematic illustration and photograph of SP‐COOH and MC‐SO_3_. b) Graphical illustrations of the SP solutions using two‐wavelength controlled patterns in a 96‐well plate. c) Schematic illustration of the SP dye‐coated TPEE film for three‐stage information encryption. d) Synthetic process of upcycled TPEE grains fabricated from recycled PET scraps.

The conceptual design of this work is depicted in **Figure** **1**. As reported by several studies,^[^ ^42^, ^43^ ^]^ SP molecules undergo a ring‐opening reaction with a heterolytic C─O bond cleavage, contributing to the formation of zwitterionic merocyanine (MC) structures. To synthesize wavelength‐dependent photochromic dyes, spiropyran derivatives with two different functional groups, SP‐COOH and MC‐SO_3_, are chosen, as depicted in Figure 1a. These two molecules, however, possess different structural changes upon light irradiations. Under UV light, SP‐COOH undergoes a ring‐opening reaction to its merocyanine state, MC‐COOH, with a solution color change from transparent to purple. The second molecule, MC‐SO_3_, changes from a merocyanine state to a ring‐closed state (SP‐SO_3_) upon UV irradiation with a solution color change from yellow to transparent. With visible light irradiations, both SP‐COOH and SP‐SO_3_ stay in their spiropyran states as transparent solutions. The opposite photochromic mechanism provides an exciting opportunity for dynamic anticounterfeiting materials in solution states, as presented in Figure 1b. SP‐COOH and MC‐SO_3_ solutions are injected into a 96‐well plate at selective regions. We select 365 nm, 555 nm, and darkness to control the structural variation between SP‐COOH and MC‐SO_3_, demonstrating different patterns in the plate. These phenomena can be reversibly controlled by applying different light conditions. This dual‐response feature is critical for achieving two‐step information concealment, a novel approach in anticounterfeiting applications. The novelty of this work lies not only in the photochromic properties of spiropyran but also in the strategic arrangement of chemical structures to control information display and concealment through light irradiation.

To further investigate the applications of opposite‐mechanism reversible spiropyran molecules, we have also applied the dyes on flexible upcycled TPEE polymer films, as displayed in Figure 1c,d. Upcycled TPEE grains are fabricated from recycled PET scraps, as described in Supporting Information (Figures S5 and S6, Supporting Information). Compared with recycled PET films, spin‐coated TPEE films possess better properties, including higher stretchability, solvent corrosive resistance, and durability, as shown in Figure 1d. The spiropyran dyes are incorporated with thickener and coated on the TPEE films with selective patterns. When exposed to different wavelengths of light, various patterns emerge, revealing encrypted information. In this work, we apply orthogonal wavelength‐controlled spiropyran designs to various scenarios, including solutions (**Figure** **3**), floral dyeing (**Figure** **4**), coatings (**Figure** **6**), and wearable substrates (**Figure** **7**). This adaptability highlights the ink's responsiveness across liquid and solid environments, enabling rapid and reversible transitions. We further validate its anticounterfeiting potential through three distinct demonstrations, achieving innovative concealment and authentication effects rarely observed in previous studies.

**Figure 2:**
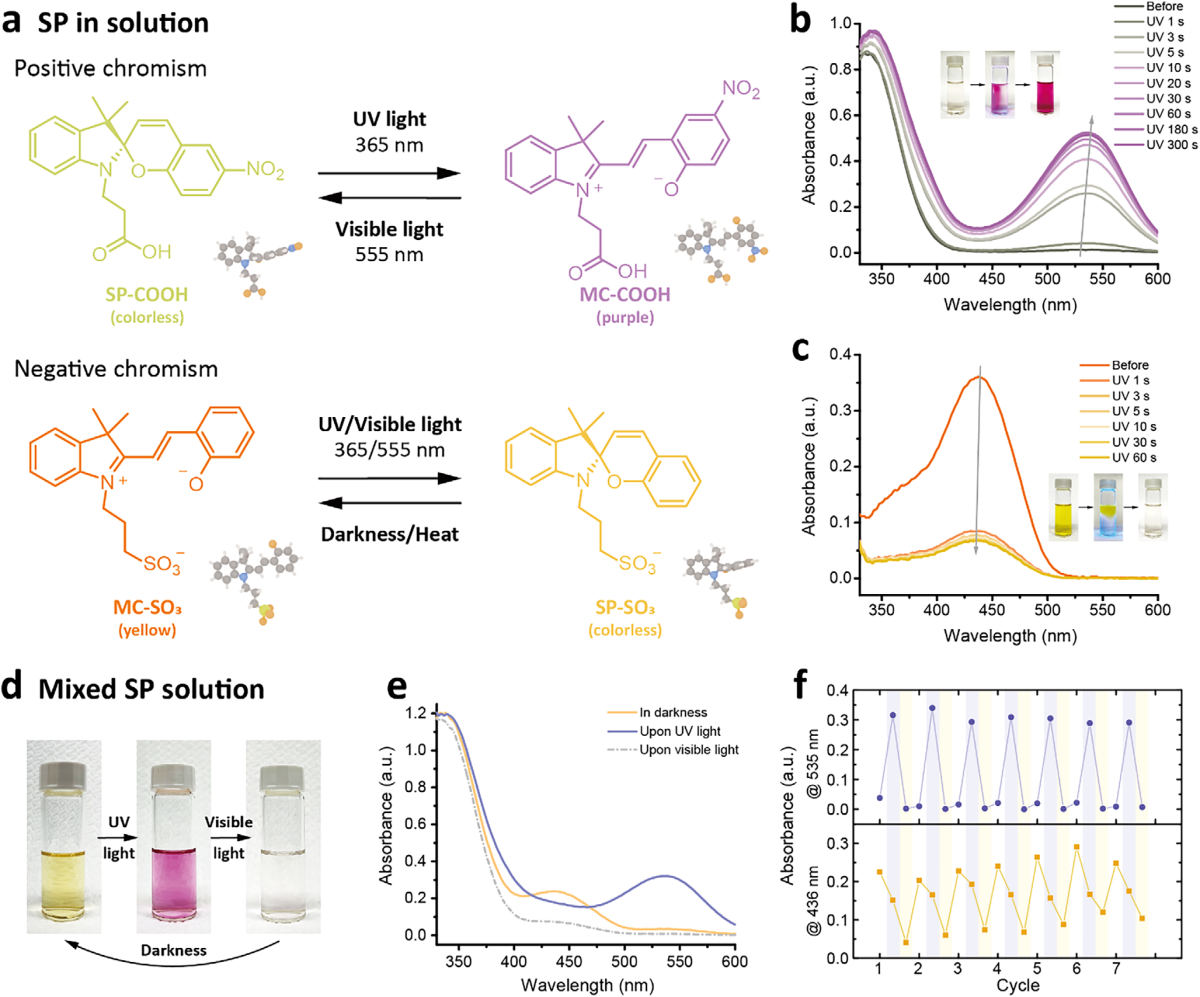
Photoswitching of the ring‐opening reactions of the SP‐COOH and MC‐SO_3_ dyes in solution. a) Reversible ring‐opening reactions of the SP derivatives. b) UV–vis absorption spectra of the b) SP‐COOH and c) MC‐SO_3_ during UV irradiations for different times. d) Photograph, e) UV–vis absorption spectra, and f) absorbance with multiple cycles of the mixed SP solution for the three‐stage color changes.

The basic photochromic properties of the SP‐COOH and MC‐SO_3_ dyes are depicted in **Figure** **2**a–c. According to previous studies, SP molecules suffer from a heterolytic C─O bond cleavage upon external stimuli, resulting in the formation of ring‐opening zwitterionic MC structures.^[^ ^42^ ^]^ For SP‐COOH, positive photochromism from colorless (SP form) to purple (MC form) is demonstrated upon UV irradiation (360 nm) and the MC form could return back to SP form under visible light (555 nm). Time‐evolved ultraviolet‐visible (UV–vis) absorption spectra and photographs of the SP‐COOH solutions are conducted to track the photoswitchable properties, as shown in Figure 2b. When exposed to UV light, the ring‐closed SP‐COOH absorbs photons and undergoes a photochemical reaction for the opening of the spiro‐ring, contributing to a new absorption peak at a wavelength of ≈535 nm owing to the π‐π* transitions in the conjugated system (Figure 2b). For MC‐SO_3_, negative photochromism, however, is achieved by functionalizing the SP structures with sulfonate groups. It has been reported that the positive charge of nitrogen on the indoline moiety can be shielded by alkyl sulfonate groups, disfavoring the nucleophilic ring‐closing reaction and stabilizing the ring‐opening merocyanine structures.^[^ ^43^ ^]^ When exposed to UV and visible lights, the ring‐opened MC‐SO_3_ undergoes a nucleophilic ring‐closing reaction to yield SP, exhibiting a decrease in the broad absorption band at a wavelength of ≈436 nm (Figure 2c).

A three‐stage photochromic solution can be demonstrated by dissolving the positive SP‐COOH and negative MC‐SO_3_ molecules into methanol, as displayed in Figure 2d. The colors of mixed SP solutions are responsive to different light irradiations—yellow initially, purple after UV irradiation, colorless upon visible light, and yellow again in darkness, which can also be tracked by UV–vis absorption curves (Figure 2e). The response times upon both UV and visible light are in seconds (less than 10 s), while the reversible reaction in darkness stands for a few seconds (≈20 s). Figure 2f depicts the reversibility and stability of the photochromic effect of the three‐stage photochromic solution by recording the absorbances at 535 and 436 nm of exposure to UV and visible lights for 30 s and stored in darkness for 45 s for seven cycles, presenting a good cyclic reversibility of the ring‐opening reaction of both the SP‐COOH and MC‐SO_3_ molecules.

**Figure 3:**
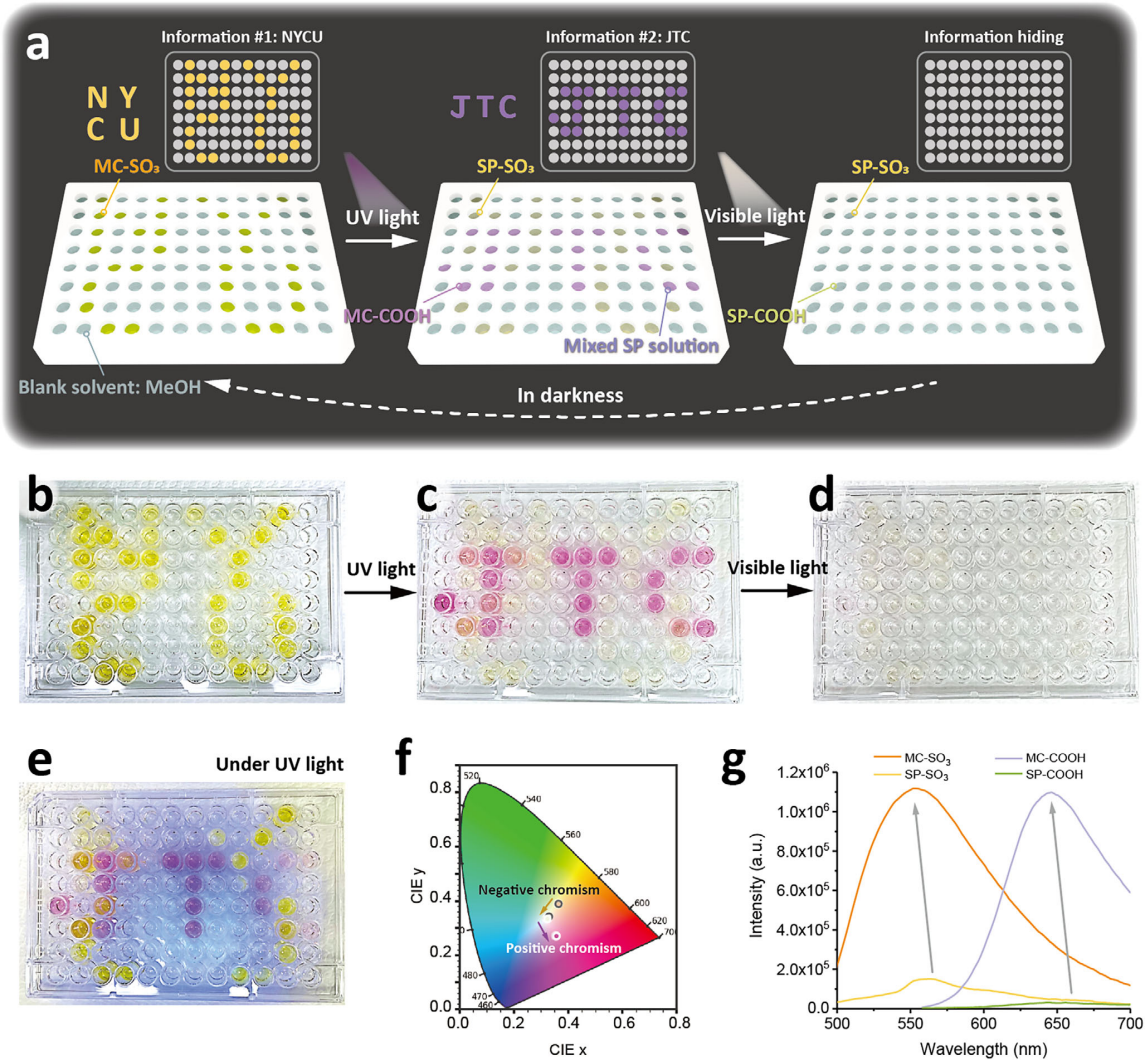
Dynamic SP solutions using two‐wavelength controlled patterns in 96‐well plates. a) Graphical scheme of the three patterns in the 96‐well plates using SP‐COOH and MC‐SO_3_. b–d) Photographs of the three patterns: b) before light irradiation, c) after UV irradiation, and d) after visible light. e) Photograph of the SP solutions under UV light. f) CIE 1931 diagrams of SP solutions. g) Fluorescence emission of SP‐COOH and MC‐SO_3_ solutions (𝜆_ex_ = 470 and 550 nm).

With reversible photoswitchable properties of the three‐stage photochromic solution, we further demonstrate a dynamic information‐revealing device by applying the SP‐COOH, MC‐SO_3_, and mixed SP solutions in a 96‐well plate, as illustrated in Figure 3a. Taking advantage of the difference in absorbances and color changes of the SP molecules, visual patterns occur simultaneously under UV and visible light irradiations. Initially, the word NYCU is displayed by applying an MC‐SO_3_ methanol solution, as shown in Figure 3b. While shining with UV light for 10 s, the “NYCU” disappears because of the ring‐closing reaction of MC‐SO_3_; subsequently, the word “JTC” occurs with a purple color, indicating the isomerization of SP‐COOH solution (Figure 3c). After shining with visible light for 10 s, the patterns, both “NYCU” and “JTC,” become invisible, revealing the spiropyran states of the SP‐COOH and SP‐SO_3_ solutions, as displayed in Figure 3d. The three‐stage photochromic anticounterfeiting solutions can be regenerated by being stored in darkness for 1 min.

With different physicochemical properties, SP and MC exhibit divergent emission behaviors, as shown in Figure 3e–g. To investigate the color changes with light treatments, we determine the colors of different stages by testing the SP‐COOH and MC‐SO_3_ in the CIE 1931 chromaticity diagram, as displayed in Figure 3f. The results show that the color differences of both positive chromism (SP‐COOH) and negative chromism (MC‐SO_3_) are detectable in low concentrations (2 mg/5 mL). As reported by previous studies,^[^ ^42^, ^43^ ^]^ ring‐opened MC molecules often exhibit an intense emission band, while the SP isomers hardly show strong emission. The photoluminescence spectra of the dyes are shown in Figure 3g, in which the luminescence peaks of the MC states are 552 and 646 nm for the MC‐SO_3_ and MC‐COOH, respectively. Their SP states, however, exhibit much lower intensities in the corresponding wavelengths, indicating that the emissions of the MC structures can be turned off because of broken π‐conjugation and pyran ring re‐forming.

**Figure 4:**
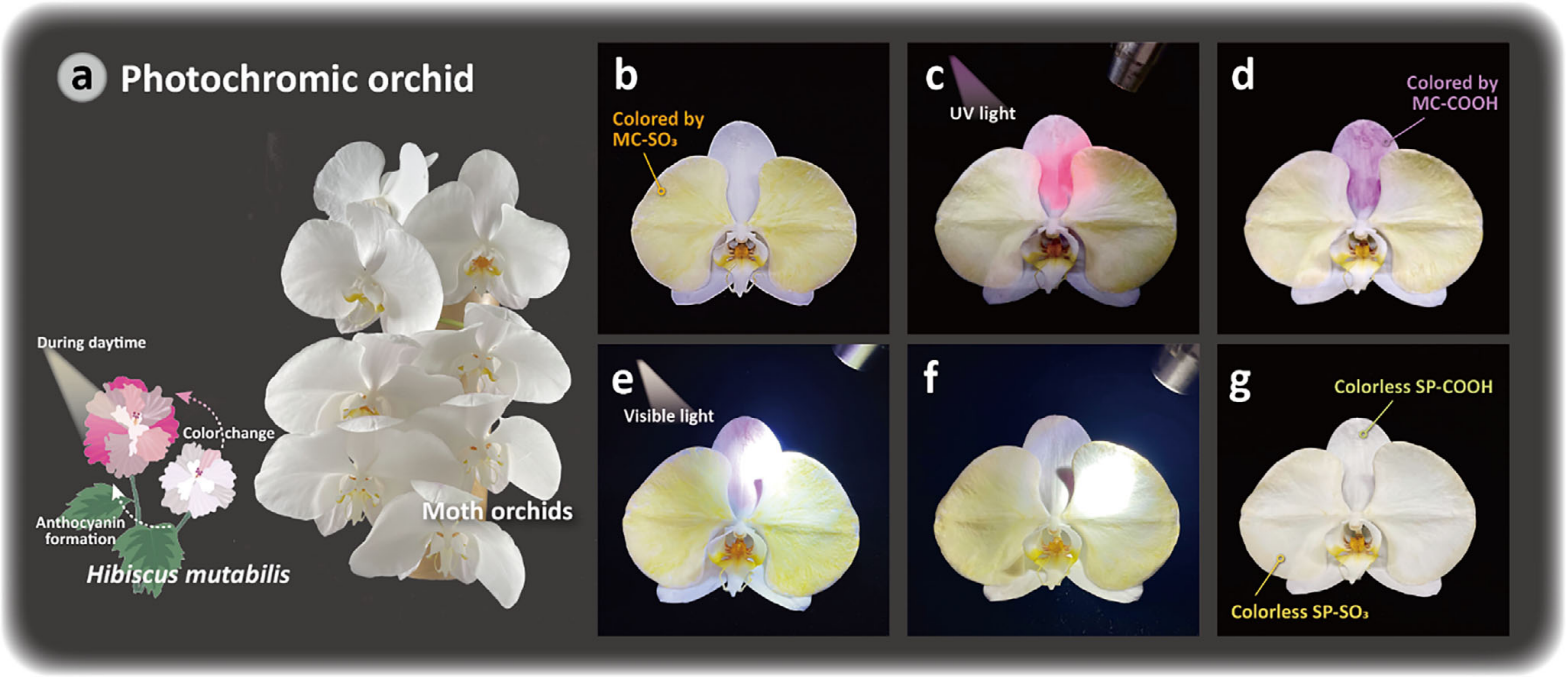
Photochromic orchids using SP dye painting with color changes upon irradiations. a) Photograph of initial white moth orchids and graphical illustration of color change of *Hibiscus mutabilis*. b–d) Photographs of the SP‐colored orchid irradiated by UV light: b) initial orchid with yellow petals, c) upon UV irradiation, d) after UV irradiation. e–g) Photographs of the SP‐colored orchid irradiated by visible light: upon visible light for e) 3 s and f) 10 s, and g) after visible light irradiation.

Besides dynamic anticounterfeiting in the solution state, the three‐stage photochromism of the SP dyes can be further applied in solid states while adding thickener, broadening their accessibility to potential applications. Inspired by the anthocyanin formation and color changing of *Hibiscus mutabilis*,^[^ ^44^ ^]^ we demonstrate the artificial light‐induced color on the petals of moth orchids. Moth orchids, as associated with elegance, virtues, and integrity, are the most well‐known topics for Chinese floral painting. In this work, white orchids are chosen and cut to be plain substrates, as shown in Figures 4a and S7, Supporting Information. Initially, two side petals of a white orchid are colored with MC‐SO_3_, presenting a light yellow color (Figure 4b), while the middle petal is painted with colorless SP‐COOH. When exposed to UV light, the middle petal with SP‐COOH dye turns purple immediately because of the exhibition of MC‐COOH, as shown in Figure 4c,d. As the negative chromism of MC‐SO_3_, the color of the side petals also becomes lighter. The yellow and purple colors on the petals can be erased by shining with visible light for 30 s, indicating the ring‐closing of the MC states and the formation of the SP molecules, as displayed in Figure 4e–g. This phenomenon can be demonstrated reversibly several times up to 15 days, which is the normal withering time of cut orchid bouquets, indicating that the painting technique does not cause much harm to the flowers, as presented in Figure S8, Supporting Information. To further compare the differences between various storage times, the color changes of the photochromic orchids over different storage durations are shown in Figure S9, Supporting Information. The brightness and color recognition of the petals remain consistent until the orchid begins to wither, demonstrating the stability of the SP‐based inks' photochromic properties and their suitability for floral painting. To our knowledge, this work is the first work that has applied light‐induced molecules to floral painting with a simple painting method, which holds potential commercial opportunity.

Moving on to the versatile applications of the three‐stage photochromism, we apply the SP dyes on upcycled TPEE materials, further upgrading the value of upcycled polymers. As the most manufactured plastic globally, PET is the main material in clothing and food packaging, which further causes severe challenges in waste management.^[^ ^7^ ^]^ To address this issue, PET wastes are selected as starting materials, undergo depolymerization and polycondensation, and are repurposed into new valuable TPEE with excellent stretchability and durability. Notably, although the depolymerization requires ≈200 °C, this temperature is well below the melting point of PET (≈250 °C) and enables efficient chemical recycling without melting the polymer. TFA is used to dissolve the TPEE segments for film formation but is fully removed during the drying process. For scale‐up, we propose screw heating and material collection as a safer and more practical alternative to spin‐coating. Meanwhile, we are also exploring greener and less hazardous solvent alternatives. The upcycled TPEE polymer possesses outstanding durability, flexibility, and sustainability, making it a promising candidate for scalable applications in robust packaging labels and anticounterfeiting tags, thereby warranting our focus on characterization and practical demonstrations.

**Figure 5:**
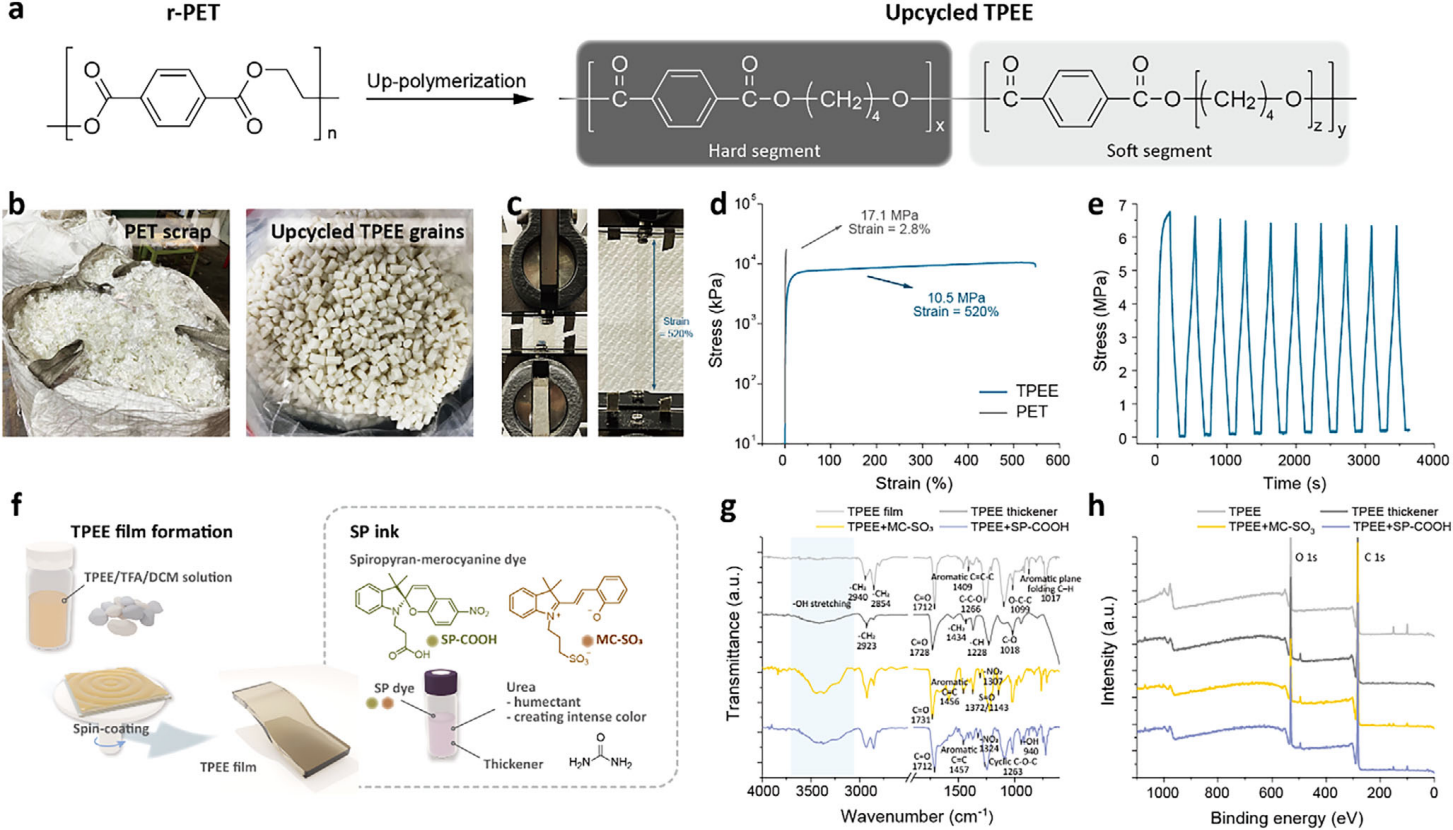
Synthetic scheme and characterizations of the upcycled TPEE and SP‐colored TPEE films. a) Synthetic process of the upcycled TPEE grains from recycled PET. b) Photographs of the PET scraps and TPEE grains. c–e) Tensile tests of the r‐PET and TPEE films. c) Photographs of the TPEE films at 0 and 550% strain. d) Stress‐strain curves of the r‐PET and TPEE films. e) Stresses during cyclic tests of the TPEE film. f) Schematic presentation of the TPEE films and SP inks. g) FT‐IR and h) XPS curves of the pure TPEE film and thickener, MC‐SO_3_, and SP‐COOH coated on a TPEE film.

The chemical structures and real images of both PET waste and TPEE are displayed in **Figure** **5**a,b, respectively. The detailed synthetic procedures of the TPEE grains are presented in Figures S5 and S6, Supporting Information. Recycled PET (r‐PET) films are collected from the residues with uneven thicknesses of the biaxially oriented polyethylene terephthalate (BOPET) films, followed by depolymerization and polycondensation processes. With a melting temperature above 250 °C, PET is typically fabricated and modified at similar temperatures, as reported in several studies on its degradation using different catalysts.^[^ ^45^, ^46^ ^]^ Figure 5d displays the stretchability performance of the PET and TPEE films. Because of its segmented copolymer structure combining hard and soft domains, the TPEE film can be stretched up to ≈520% strain (Figure 5c), whereas the limited strain of the pure PET film (≈2.8%) is likely due to processing conditions and the absence of plasticizers. The cyclic tests at the stress ≈6.5 MPa also show stability for 10 cycles, indicating good stability under tension, as shown in Figure 5e. Figure S10, Supporting Information shows the cyclic stress–strain curves for 50 cycles under 500% strain, which illustrate the mechanical properties and recovery behavior of the upcycled TPEE films, indicating their stretchability and durability for repeated use. From a practical standpoint, we have also scaled up production in a 200 L reactor, which processes significantly larger amounts (on the kg scale) compared to lab‐scale fabrication. Although PTMEG greatly improves the stretchability of upcycled TPEE, a simple film format may not fully showcase its benefits. We are thus advancing toward fabric‐based materials, where their enhanced mechanical properties offer strong potential for scalable, cost‐effective applications.

After the fabrication of the TPEE films, a mixture of the MC‐SO_3_ and SP‐COOH dyes, thickener, and urea is prepared for better adhesion, as presented in Figure 5f. The upcycled TPEE films exhibit good interfacial compatibility with the spiropyran‐based coating, with hydrogen bonding in the soft segments and moderate surface hydrophilicity contributing to uniform and durable adhesion without dewetting. Figure S11, Supporting Information shows the water contact angles of the upcycled TPEE and the SP ink‐coated TPEE films. The chemical structures of the pure TPEE film and thickener, MC‐SO_3_, and SP‐COOH coated on TPEE films are confirmed by Fourier‐transform infrared spectroscopy (FT‐IR), as shown in Figure 5g. The broad absorption peaks from 3000 to 3700 cm^−1^ are for the O‐H bending in the thickener. With the TPEE films as substrates, the absorption peaks appearing at 2940, 2854, and 1712 cm^−1^ are attributed to the stretching vibration of C─H, ─CH_2_, and C═O, respectively, which can also be observed in the thickener and SP dyes coated TPEE films. The two absorption peaks at 1372 and 1143 cm^−1^ are characteristic peaks for S = O stretching, which can be assigned to the sulfonate of the MC‐SO_3_. It can also be observed that an absorption peak at 940 cm^−1^ contributes to ‐OH bending in the carboxyl group of the SP‐COOH. Meanwhile, the absorption peaks at 1307 and 1324 cm^−1^ are attributed to ‐NO_2_ groups in MC‐SO_3_ and SP‐SOOH, respectively.

X‐ray photoelectron spectroscopy (XPS) is conducted to characterize the elemental compositions of the sample surfaces, as shown in Figure 5h. On the whole, the TPEE, thickener, MC‐SO_3_, and SP‐COOH all contain C and O elements, which can be seen at the peaks at 283.2 and 530.6 eV, respectively. Because of the thicker layer of the thickener and less amount of the SP dyes, the S and N elements do not show significant signals for the samples coated with MC‐SO_3_ and SP‐COOH. The increased amounts of the N elements in both SP dyes and S elements in MC‐SO_3_ can be observed in the elemental compositions of the samples, as listed in Table S1, Supporting Information. For the cost of the materials, the estimated costs of upcycled and petrochemical‐based TPEE are provided, as listed in Table S2, Supporting Information. The total cost of upcycled TPEE is estimated at $3.65 kg^−1^—lower than conventional TPEE's $4.11 kg^−1^—based on comparable processing expenses and the use of recycled PET and recoverable reagents.

**Figure 6:**
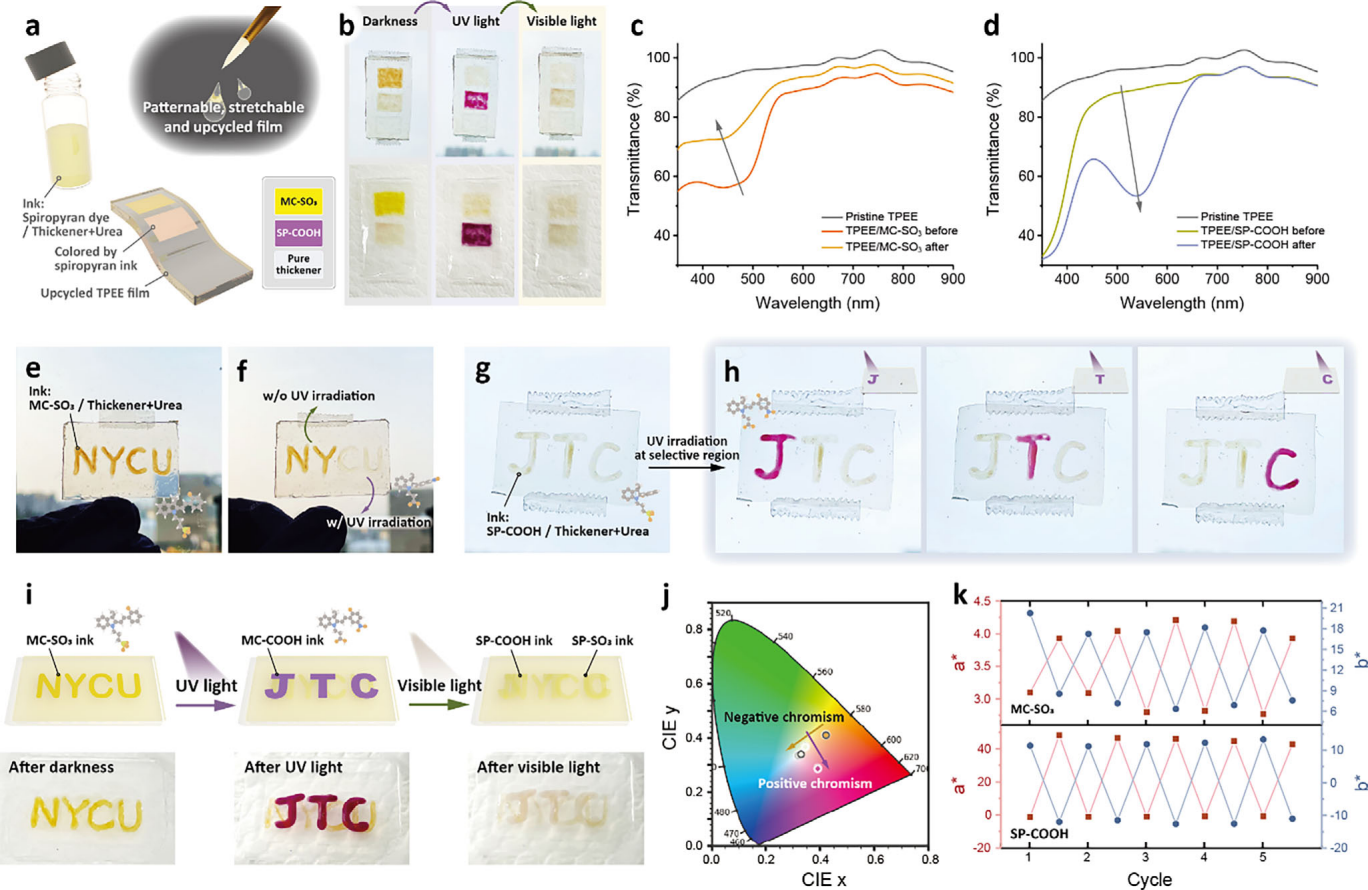
Photoswitchable chromism under two‐wavelength irradiation and anti‐counterfeiting application. a) Graphical illustration of the fabrication of the SP color card using a TPEE film. b) Color changes of the SP color card under different light irradiations. c,d) UV–vis transmittance spectra of the c) MC‐SO_3_ and d) SP‐COOH coated TPEE films compared with pure TPEE films before and after UV irradiations. e,f) MC‐SO_3_ ink‐coated TPEE film with an “NYCU” pattern e) before and f) after UV irradiations at selective regions. g,h) SP‐COOH ink‐coated TPEE film with a “JTC” pattern g) before and h) after UV irradiations at selective regions. i–k) Information encryption applications using two‐wavelength controllable inks. i) Graphical illustrations and photographs of the samples. j) CIE 1931 diagrams and k) cyclic data of SP‐coated TPEE films.

After applying SP inks on the upcycled TPEE films, the patternable and stretchable materials with photochromic properties can be demonstrated, as shown in Figure 6a. To track the color changes between different light irradiations, a color card with MC‐SO_3_, SP‐COOH, and pure thickener has been fabricated. Figure 6b displays the photographs of the color card with three regions. Initially, the region of MC‐SO_3_ exhibits a yellow color, while the regions of SP‐COOH and pure thickener are colorless. After being irradiated with UV light for 30 s, the region of MC‐SO_3_ turns out to be colorless with the ring‐closing of the MC forms; the region with SP‐COOH, surprisingly, becomes purple because of the appearance of the MC‐COOH. Upon visible light irradiation, the colors on both regions of the MC‐SO_3_ and SP‐COOH disappear, indicating the SP states of the dyes. To investigate the color changes of the films, UV–vis transmittance spectra of the films are displayed in Figure 6c,d. Compared with pure TPEE films, the MC‐SO_3_ coated films exhibit a transmittance peak at 440 nm, resulting in a yellow color, which is similar to the trend for MC‐SO_3_ solutions (Figure 6c). The intensity of the peak at 440 nm decreases after being shone with UV light. The SP‐COOH coated TPEE films, interestingly, show an increase of a peak at 540 nm after UV irradiation, demonstrating the same results as the SP‐COOH solutions (Figure 6d).

To deepen the applications of the upcycled TPEE films with SP dyes, the MC‐SO_3_ and SP‐COOH inks are applied to the films in selective regions with ink brushes. Figure 6e shows the transparent TPEE film with a pattern of “NYCU” colored by the MC‐SO_3_ ink. Subsequently, the film is shone by UV light at the selective region (the word “CU”) for 30 s to generate the photoisomerization, as displayed in Figure 6f. The TPEE film with a pattern of “JTC” colored by SP‐COOH ink is displayed in Figure 6g. Initially, the pattern “JTC” is colorless based on the colors of the thickener and SP‐state dyes. By shining UV light at selective regions, the words “J,” “T,” and “C” can be highlighted by turning to purple color with the MC states of SP‐COOH. After 30 days of storage, the color changes of the MC‐SO_3_ and SP‐COOH ink‐coated TPEE films remain observable, as shown in Figures S12 and S13, Supporting Information, indicating the stability of the materials.

In general, the photochromisms of the SP dyes are efficient and distinguishable on the TPEE films, inspiring us for the information encryption by the different reactions upon light irradiations. Figure 6i shows the graphical illustrations and photographs of the anticounterfeiting samples. The yellow word “NYCU” can be seen first just after being stored in darkness. The sample is then shone with UV light for 30 s to generate the isomerization from MC‐SO_3_ to SP‐SO_3_ and from SP‐COOH to MC‐COOH, causing the disappearance of the word “NYCU” and the appearance of the word “JTC,” respectively. After being shone with visible light for 30 s, the words “NYCU” and “JTC” become colorless because of the SP states of the dyes. This phenomenon of color changes provides a new opportunity to involve and reveal information with proper light irradiations. The colors of the SP dyes on the uncycled TPEE films are also examined by the CIE 1931 diagram, as shown in Figure 6j. To observe the possible photo fatigue effects of the SP molecules, as reported in previous studies,^[^ ^47^ ^]^ we further track the color changes upon light irradiations. Figure 6k presents the results during cyclic tests of SP‐coated TPEE films in the CIE 1931 diagrams, where the a* and b* are the red/green coordinate and yellow/blue coordinate, respectively. Similar to the results in the UV–vis absorbance of the SP solutions, there is no noticeable decrease in the color performance after repeating for 5 cycles, demonstrating the good stability of SP‐coated upcycled TPEE films.

**Figure 7:**
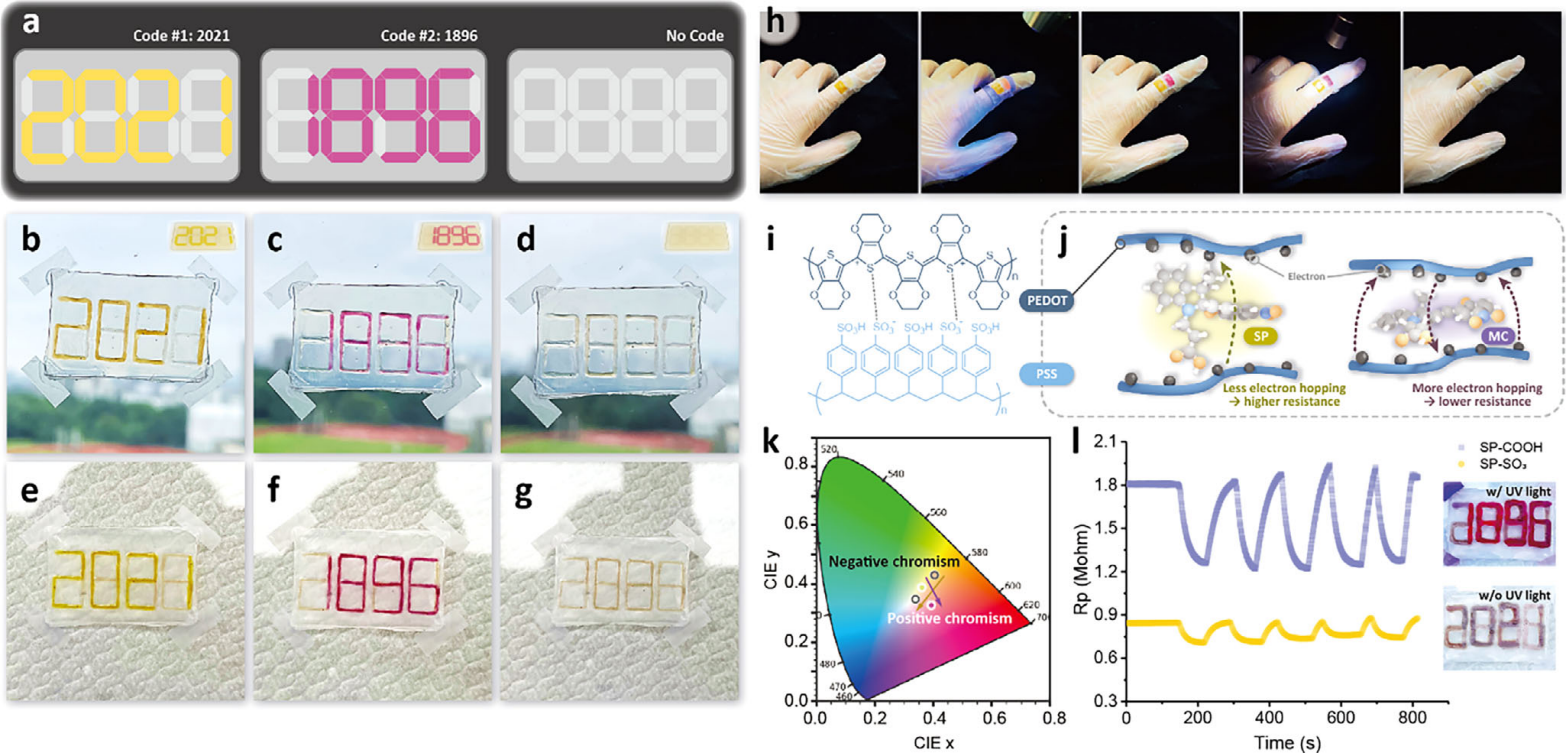
Versatile anti‐counterfeiting applications of the SP‐coated upcycled TPEE films. a) Graphical illustration of different codes upon light irradiations. b–g) Photographs of different visible codes (b,e) initially, (c,f) after UV irradiations, and (d,g) after visible light irradiations. h) Photographs of flexible SP‐coated upcycled TPEE films as wearable devices. i) Chemical structures and j) mechanism of PEDOT: PSS incorporated samples. k) CIE 1931 diagrams of the PEDOT: PSS incorporated samples. l) Electrochemical impedance spectra of the PEDOT: PSS incorporated samples for different cycles.

By patterning digital numbers, the information hidden on the TPEE films can be more consistent, as shown in Figure 7a–g. The color changes upon different light irradiations can be distinguished in both transmission states and with a backboard. Meanwhile, taking advantage of the stretchable TPEE, the films can be attached to gloves, showing the potential applications on wearable devices (Figure 7h). The UV and visible light responses of the SP‐coated TPEE films are demonstrated under certain tensions, such as finger bending. After storage for 180 days (Figure S14, Supporting Information), the stretchability and stability remain good compared with the original TPEE films (Figure 5e). The slight differences between the MC‐SO_3_ and SP‐COOH coated TPEE films are mainly due to the different ink coating thicknesses on the films, which affect the results when calculating the stress relative to the cross‐sectional area of the films. TPEE was selected for its balance of elasticity, strength, and stretch recovery, making it especially suitable for durable and flexible devices. The results highlight the upcycled TPEE films as promising flexible and stable materials, offering deeper insight into their durability for wearable applications.

As a candidate for soft electronics, poly(3,4‐ethylenedioxythiophene) polystyrene sulfonate (PEDOT:PSS) is a well‐known commercial conductive polymer pair.^[^ ^48^, ^49^ ^]^ Figure 7i displays the chemical structure of the PEDOT:PSS polymer. As reported by our previous work,^[^ ^50^ ^]^ the conductivity of PEDOT:PSS highly corresponds to the stacking distances between the PEDOT polymer chains, which can be increased by controlling the 3D structures of the SP and MC molecules, as shown in Figure 7j. Under UV light, the SP undergoes a ring‐opening reaction, transitioning from its 3D molecular structure to the planar 2D structure of MC. With the non‐planar 3D structure of the ring‐closed SP molecules, the stacking distances between the PEDOT chains are assumed to increase, reducing the charge transfer efficiency and leading to higher resistance. While being shone with UV light, the generated planer MC molecules can decrease the stacking distances, allowing PEDOT chains to stack more closely, facilitating electron hopping and improving charge transfer efficiency. Therefore, the conductivity of PEDOT:PSS films can be modulated by light through the reversible ring‐opening and closing reactions of SP and MC, which alter the stacking distances of PEDOT chains and affect their charge transfer efficiency. The color of the PEDOT:PSS, however, is dark blue, limiting the efficiency of the photochromic anticounterfeiting; therefore, in this work, we add a minimum amount of the PEDOT:PSS for ≈20 mg to enhance the conductivity of the samples, which only grays out the colors of the SP inks (Figure 7k). Electrochemical impedance spectra of the PEDOT:PSS incorporated samples for different cycles are presented in Figure 7l. The resistance changes of the MC‐SO_3_ and SP‐COOH inks on the TPEE films can be observed during cycles of UV and visible lights. The initial resistance of the SP‐COOH ink is ≈1.8 MΩ, while the resistance quickly drops to ≈1.25 MΩ after UV irradiation for 150 s. For the MC‐SO_3_ ink on the TPEE films, the resistance decreases to ≈0.7 MΩ with a pristine resistance of ≈0.85 MΩ. Furthermore, the cyclic tests of the MC‐SO_3_ and SP‐COOH inks on the TPEE films indicate photo‐controllable properties in solid states such as polymeric films, as well as the stability and reversibility of the samples, providing a second lock of the anticounterfeiting materials.

## 3 Conclusions

In conclusion, we introduce a sustainable and innovative approach to upcycling TPEE films from recycled PET waste and incorporating orthogonal wavelength‐controlled SP derivatives (SP‐COOH and MC‐SO_3_) to achieve dynamic anticounterfeiting and photoswitchable conductivity. The upcycled TPEE films demonstrate enhanced mechanical properties, including superior stretchability, solvent resistance, and durability. The spiropyran‐based materials exhibit reversible photochromic behaviors, enabling efficient information encryption through distinct visual patterns and photoswitchable conductivities when combined with conductive PEDOT:PSS. Our findings showcase the potential of utilizing upcycled polymers for advanced functional applications, addressing both environmental concerns and the need for robust anticounterfeiting technologies. This work pioneers the use of upcycled TPEE films with photoswitchable spiropyrans, offering a promising pathway for the development of sustainable materials in the fields of polymer upcycling, wearable devices, and secure information encryption.

## 4 Experimental Section

### Materials

Recycled polyethylene terephthalate (r‐PET) wastes were obtained from CHC Technology Inc (grade <100 ppm). Polytetramethylene ether glycol (PTMEG) with an *M*_w_ of 1000 g mol^−1^ and chitosan were bought from Sigma‐Aldrich. Butanediol (BDO) was purchased from Alfa Aesar.

Dichloromethane (>99.9%) was bought from Honeywell. Anhydrous ethanol (99.5%) and acetone (99.5%) were purchased from Echo Chemical. Deionized water was obtained from the Milli‐Q system (18.4 MΩ). All the chemicals were used without further purification. Microscope glass slides were obtained from Dogger Scientific (DGS). The detailed synthetic procedures of the spiropyran dyes and TPEE are listed in Figures S1–S6, Supporting Information).

### Dynamic Anticounterfeiting of SP Solutions in 96‐Well Plates

Spiropyran derivatives (1 mg for SP‐COOH and 3 mg for MC‐SO_3_) were separately added to 20 mL of methanol at room temperature. For the mixed SP solution, MC‐SO_3_ and SP‐COOH solutions with a ratio of 3:1 were used. After the solution preparation, a 96‐well plate was filled with the MC‐SO_3_, SP‐COOH, and mixed SP solutions at selective regions, while methanol was a blank solvent at the other regions.

### Fabrication and Tensile Tests of TPEE Films

2.4 g of TPEE grains, 6.91 mL of dichloromethane (DCM), and 2.96 mL of trifluoroacetic acid (TFA) were first mixed in a glass vial, followed by being stirred overnight at room temperature. After the preparation of the homogeneous TPEE solution, glass substrates with sizes of 3 × 1.5 cm^2^ were placed in a spin coater for further coating process. The TPEE solution was dropped on the glass substrates with a rotation speed of 500 rpm for 60 s, and the rotation speed was changed to 2000 rpm for another 30 s. The TPEE‐coated glass substrates were then placed in a vacuum for 3 h to remove the solvent. Finally, the TPEE films were successfully fabricated after being peeled off from the substrates. We recognize the potential toxicity concerns of TFA and have explored process modifications. Instead of using spin‐coating, which is suitable for lab‐scale film formation but not for large‐scale production, we propose screw heating followed by material collection after cooling, offering a safer, more efficient, and scalable alternative.

For the tensile tests, pieces of TPEE films and PET films were cut into 1 × 3 cm^2^ and loaded onto a tensile test machine. The measurement was conducted with a 5 mm min^−1^ displacement rate at room temperature for uniaxial elongation.

### Preparation of SP‐Based Inks and SP/PEDOT:PSS Inks

For the preparation of SP‐COOH ink, 70 mg of SP‐COOH and 0.6 mL of dimethylformamide (DMF) were mixed in a glass vial and stirred for 30 min. For the preparation of MC‐SO_3_ ink, 10 mg of MC‐SO_3_ and 0.75 mL of deionized water were mixed in a glass vial and stirred for 30 min. Mixtures of urea (0.15 g) and commercial thickener (10 g) were then added to the vials with SP‐COOH and MC‐SO_3_ inks. For the addition of PEDOT:PSS, 3 g of the SP‐COOH and MC‐SO_3_ inks were separately added with 20 mg of PEDOT:PSS and stirred at room temperature until thorough dissolution. The demonstration involving hand motion (Figure 7) was performed with voluntary participation and written informed consent. No identifiable personal data were collected, and the procedure posed minimal risk.

### Photochromic Orchids using SP Dye Painting

A bunch of moth orchids was cut and placed in a glass bottle with water. A single bloom of the orchids was selected to be painted by the photochromic SP inks. Two side petals were colored with the yellow MC‐SO_3_ ink, and the middle petal was painted with the colorless SP‐COOH ink. For the photochromism tests, the orchid was shone with UV light for 20 s, followed by alternating to visible light for 30 s in a single cycle. The preservation of the bunch of moth orchids without painting was ≈15 days. The photochromism tests were conducted to check the color changes in the 15 days before the orchids withering.

### Structure Characterizations and Analyses

An ^1^H nuclear magnetic resonance (NMR) spectrometer (JOEL 400 MHz) was applied to characterize the upcycled TPEE, synthesized SP‐COOH, and synthesized MC‐SO_3_. A Fourier‐transform infrared (FT‐IR) spectrometer (PerkinElmer Spectrum One) was conducted to confirm the chemical structures of the samples. Ultraviolet‐visible (UV–vis) absorbance and transmittance spectra were collected from 330 to 600 nm and from 350 to 900 nm, respectively, by a Hitachi U‐4100 spectrometer. CIE 1931 diagrams were measured using a spectrophotometer (Konica Minolta, CM‐5). The photoluminescence tests of the SP solutions were conducted by a spectrofluorometer (FluoroMax, Horiba). A tensile testing machine (Shimadzu, EZ) was used to measure mechanical properties. A high‐resolution X‐ray photoelectron spectrometer (HRXPS) (PHI Quantera II) was applied to confirm the elemental compositions. For the measurements of resistance changes, an LCR meter (LCR‐6000, GWInstek) was used with and without UV irradiations over a frequency of 1 kHz and a voltage of 1 V.

## Conflict of Interest

The authors declare no conflict of interest.

## Supporting information



Supporting Information

## Data Availability

The data that support the findings of this study are available from the corresponding author upon reasonable request.
