# Supplementary material for: Hibiscus Mutabilis‐Inspired Upcycled TPEE Films with Orthogonal Wavelength‐Controlled Spiropyrans for Dynamic Anticounterfeiting and Photoswitchable Conductivity
Source: Small. 2025 Jul 22;21(36):e03829. doi: 10.1002/smll.202503829 (PMC12423904; doi:10.1002/smll.202503829)
Supplement: Supplementary file 1 — Supporting Information [file SMLL-21-e03829-s001.docx]

**Supporting Information**

*Hibiscus Mutabilis*-Inspired Upcycled TPEE Films with Orthogonal Wavelength-Controlled Spiropyrans for Dynamic Anticounterfeiting and Photoswitchable Conductivity

Yi-Fan Chen,^1^ Lin-Ruei Lee,^1^ Ming-Hsuan Chang,^1^ Huan-Wei Lin,^1^ Yu-Chun Liu,^1^ Chun-Ting Chang,^1^ Kai-Chuan Kuo,^1,2^ Chun-Chi Chang,^1^ Che-Tseng Lin,^2^ and Jiun-Tai Chen^1,3^*

^1^Department of Applied Chemistry, National Yang Ming Chiao Tung University, 300093 Hsinchu, Taiwan

^2^Department of Performance Materials Synthesis & Application Division of Polymer Research Material and Chemical Research Laboratories, Industrial Technology Research Institute, 300044 Hsinchu, Taiwan

^3^Center for Emergent Functional Matter Science, National Yang Ming Chiao Tung University, 300093 Hsinchu, Taiwan

**Synthesis of Spiropyran Derivative 1 (MC-SO_3_)**

The **s**piropyran derivative 1 (MC-SO_3_) was synthesized according to a literature method.^1^ First, 2,3,3-trimethylindolenine (1.65 g, 0.01 mmol) was added to propane sultone (1.26 g, 0.01 mmol). The mixture was stirred at 90 °C for 4 h under N_2_ atmosphere. The purple solid was collected by filtration, washed with cold diethyl ether, and dried in a vacuum. Then, 2,3,3-trimethyl-1-(3-sulfonatepropyl)-3H-indolium (100 mg, 0.36 mmol) and 2-hydroxybenzaldehyde (48 mg, 0.39 mmol) were added to anhydrous ethanol (2 mL). The mixture was refluxed overnight under N_2_ atmosphere. The orange solid was then collected by filtration.

**
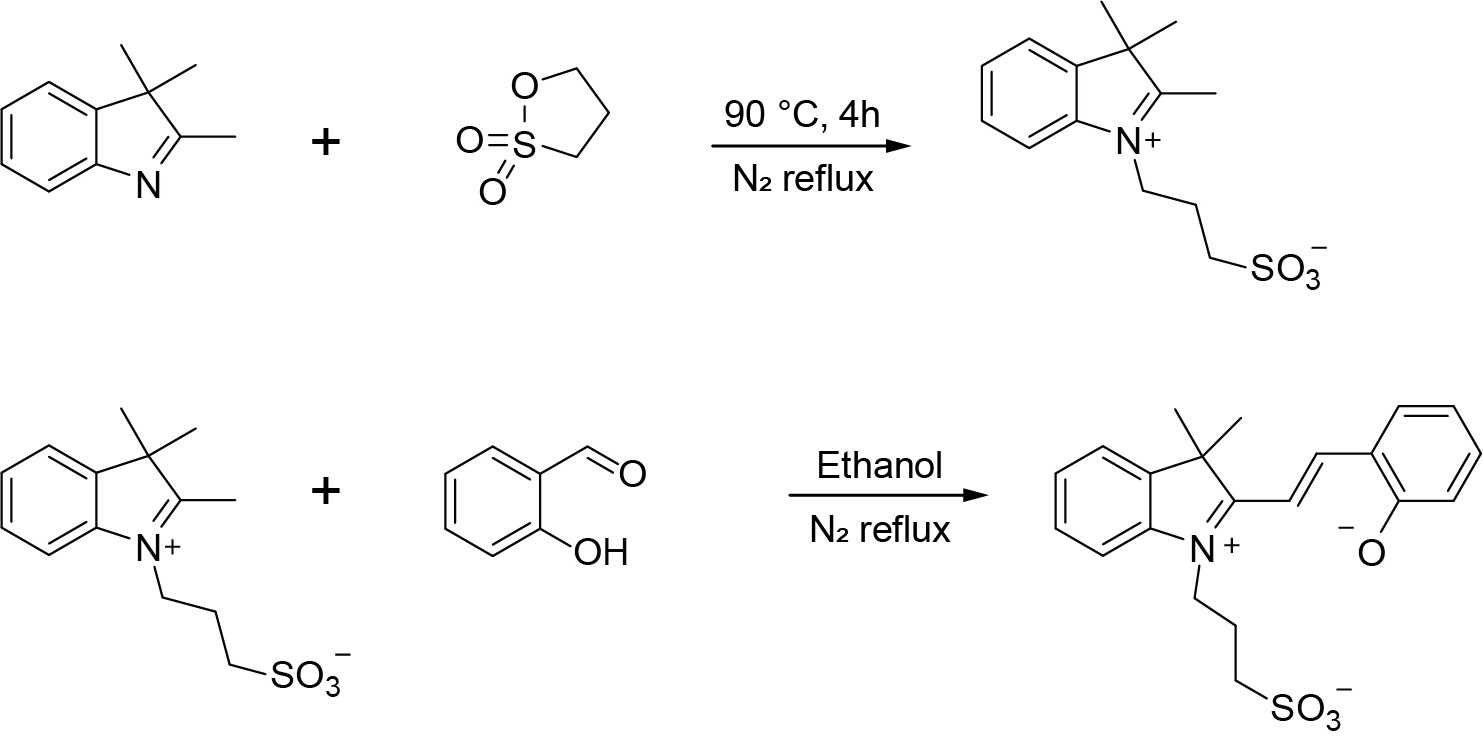
**

**Figure S1.** Synthetic scheme of the spiropyran derivative 1 (MC-SO_3_).


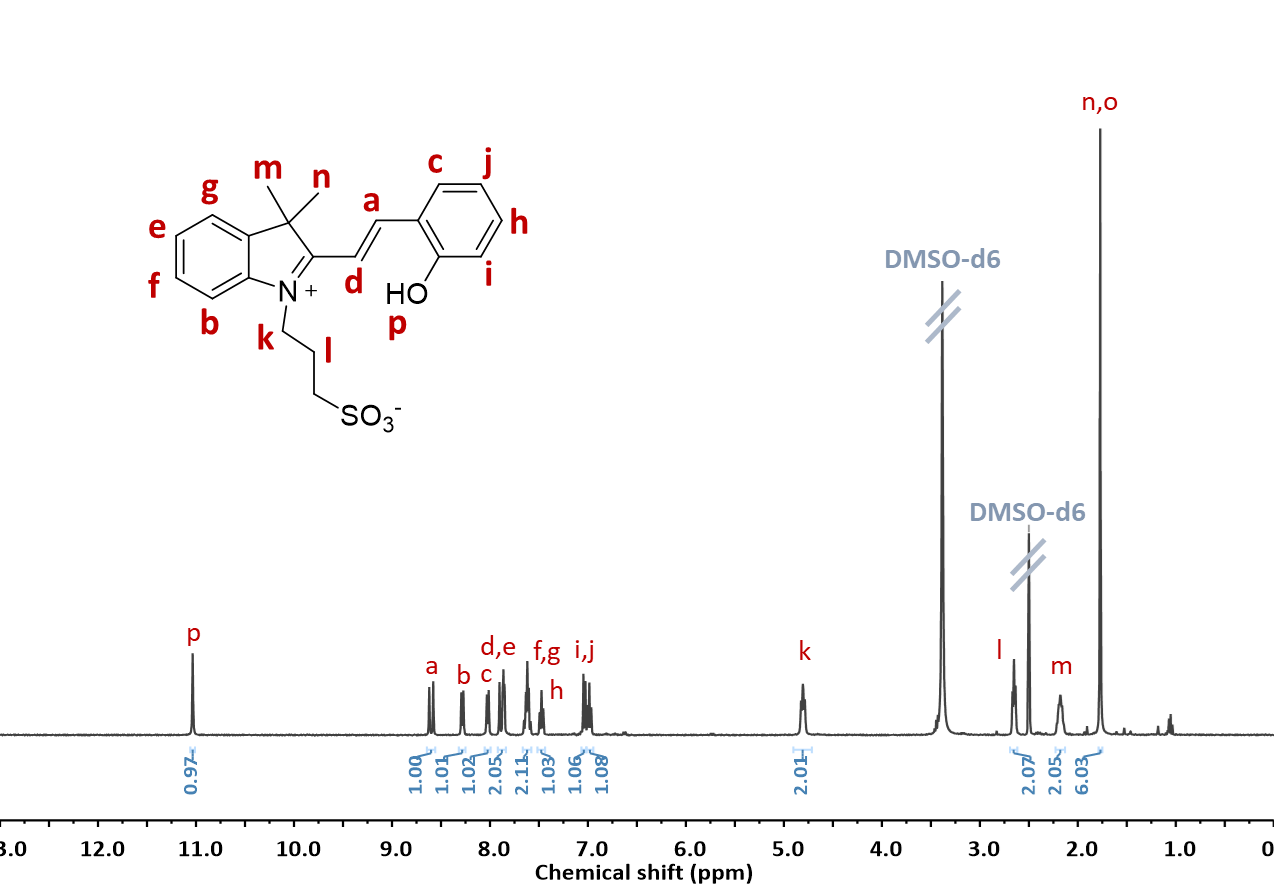


**Figure S2.** ^1^H NMR spectrum (400 MHz, DMSO-d6, 298 K) of MC-SO_3_.

**Synthesis of Spiropyran Derivative 2 (SP-COOH)**

The **s**piropyran derivative 2 (SP-COOH) was prepared following a previously reported method with some modifications.^2, 3^ First, a mixture of 2,3,3-trimethylindolenine (4.0 g, 25 mmol) and 3-iodopropionic acid (5.4 g, 27 mmol) in toluene (10 mL) was stirred and refluxed for 12 h under N_2_ atmosphere. The reaction mixture was then filtered at room temperature, and the resulting precipitate was washed several times with cold hexane and diethyl ether. The obtained intermediate, an iodide salt, was dried under a vacuum. Next, the iodide salt (4.5 g, 12.6 mmol), 5-nitrosalicylaldehyde (2.1 g, 12.6 mmol), and piperidine (1.3 mL, 13.2 mmol) were placed in a round-bottom flask. This mixture was stirred and refluxed for 5 h, followed by stirring at room temperature for an additional 5 h. After the reaction was complete, the mixture was cooled to 0 °C. The light green precipitate, SP-COOH, was obtained by filtering the mixture and washing the precipitate several times with cold ethanol.


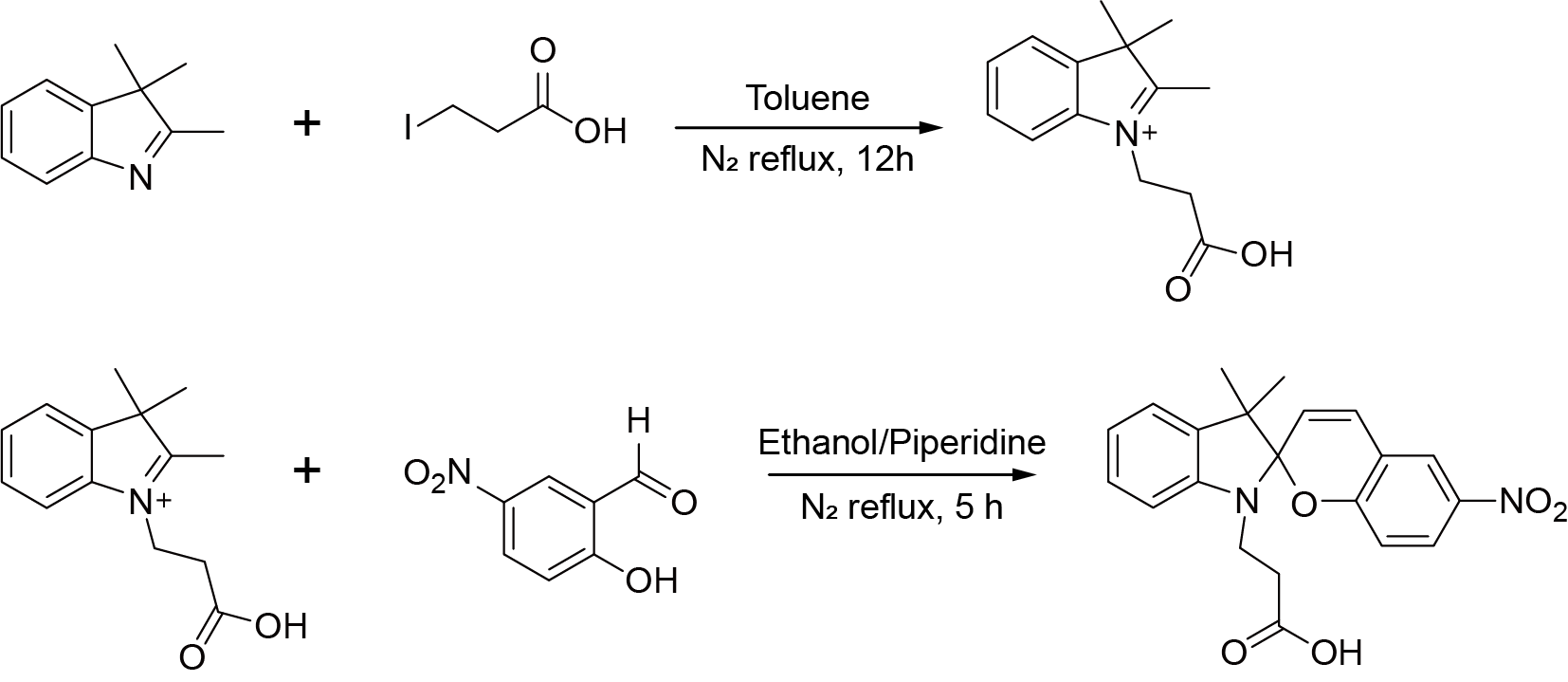


**Figure S3.** Synthetic scheme of the spiropyran derivative 2 (SP-COOH).


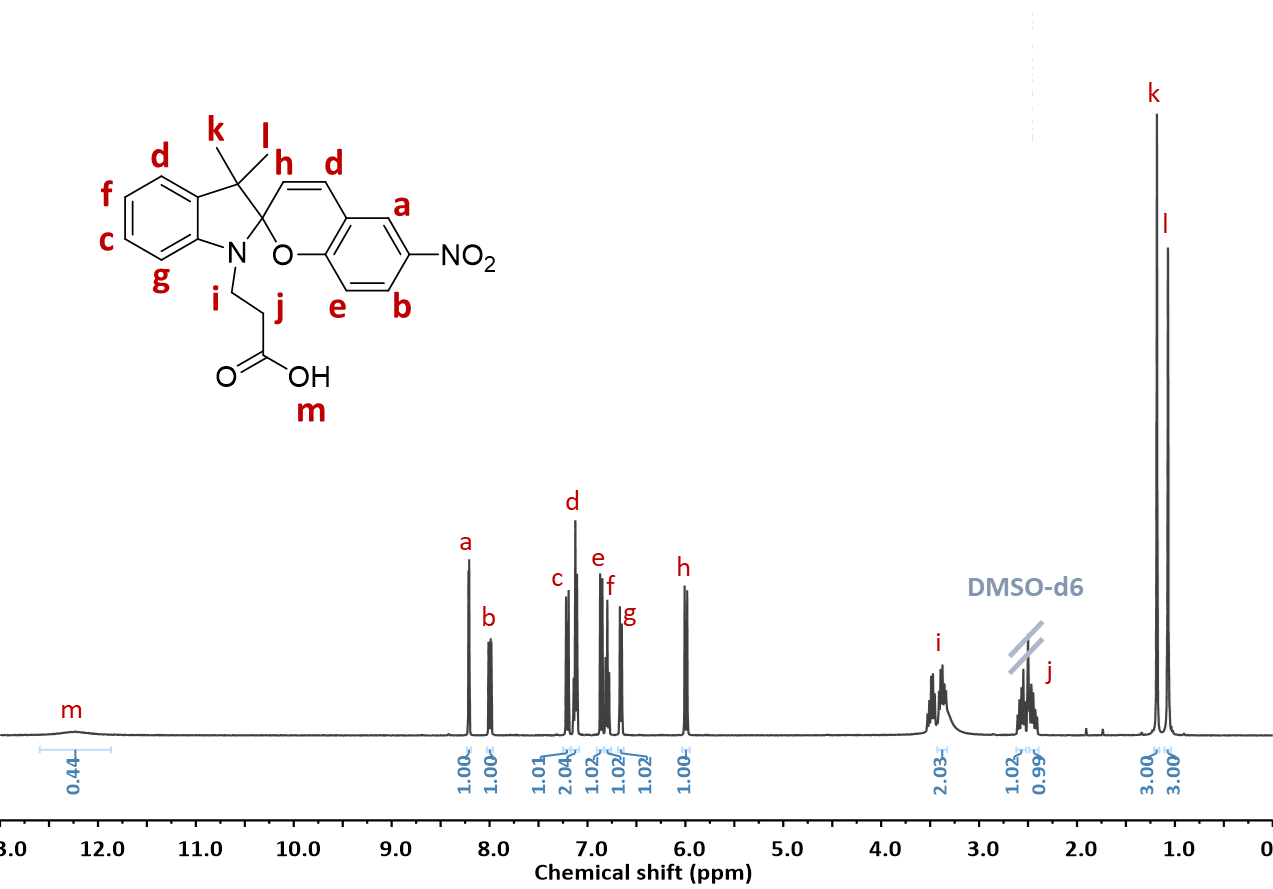


**Figure S4.** ^1^H NMR spectrum (400 MHz, DMSO-d6, 298 K) of SP-COOH.

**Synthesis of Thermoplastic Polyester Elastomer (TPEE)**

To synthesize upcycled TPEE, we employed a two-step process: depolymerization and polycondensation, as depicted in Figure S5. During depolymerization, recycled PET (r-PET), butanediol, and a titanium catalyst were introduced into a reactor. The r-PET films, sourced from biaxially oriented polyethylene terephthalate (BOPET) films, were predominantly used in Polaroid films. Residual PET films with uneven thicknesses were collected for the depolymerization process. These r-PET films were pulverized into fragments and granulated to produce r-PET granules. The TPEE grains were then fabricated from these granules through the up-polymerization and granulation processes. The mixture underwent depolymerization in a nitrogen environment at 200−220 °C for 4 h. Subsequently, polytetramethylene ether glycol, an antioxidant, and a catalyst were added to the reactor for polycondensation, which occurred at 245 °C under vacuum. The resulting TPEE solution was then cooled with water and granulated to produce upcycled TPEE grains. The hard and soft segments in TPEE were polybutylene terephthalate and polytetramethylene ether glycol, respectively.


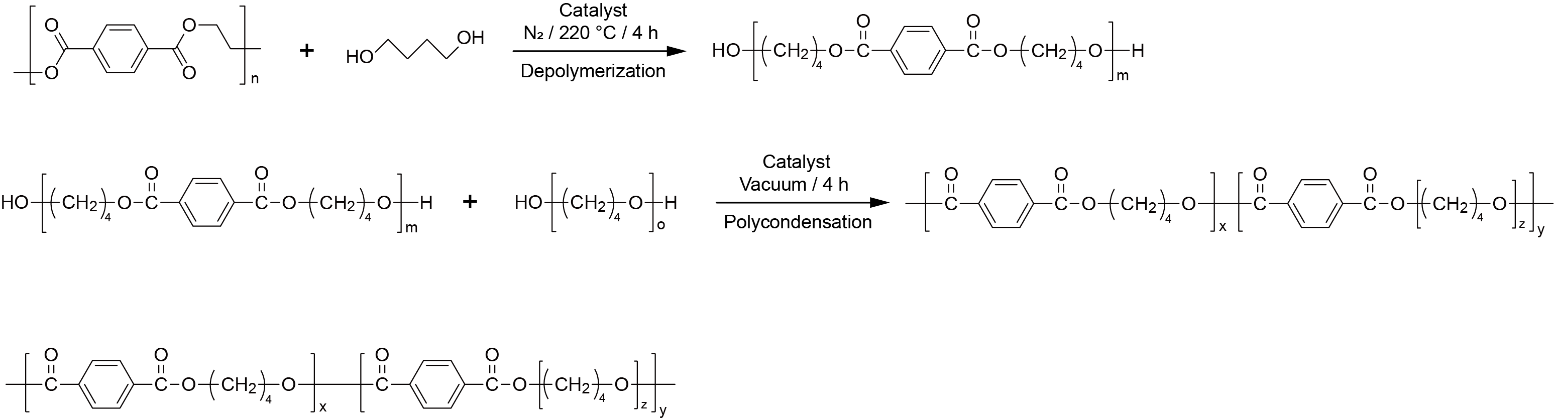


**Figure S5.** Synthetic scheme of upcycled TPEE.


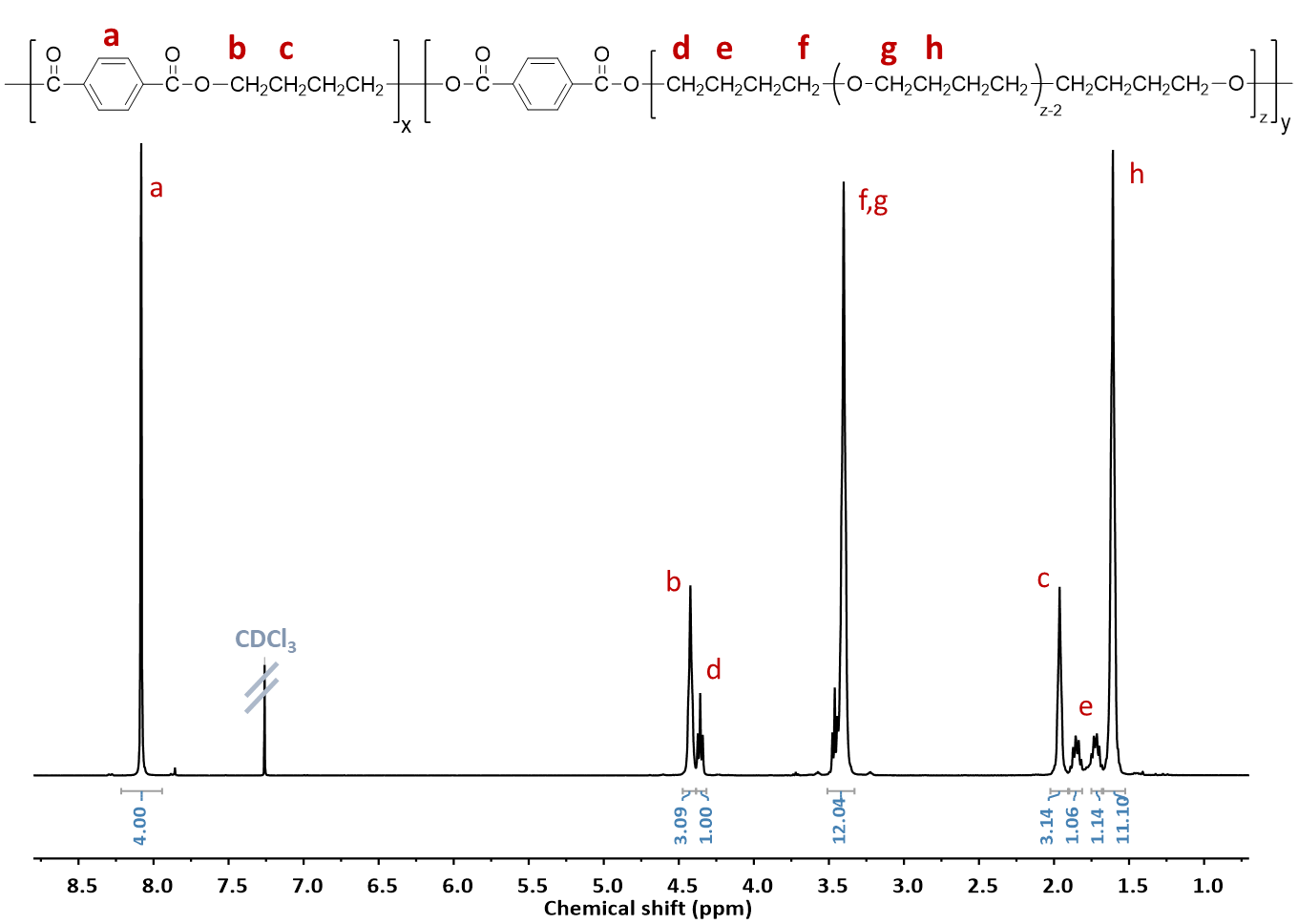


**Figure S6.** ^1^H NMR spectrum (400 MHz, CDCl_3_, 298 K) of TPEE.

**
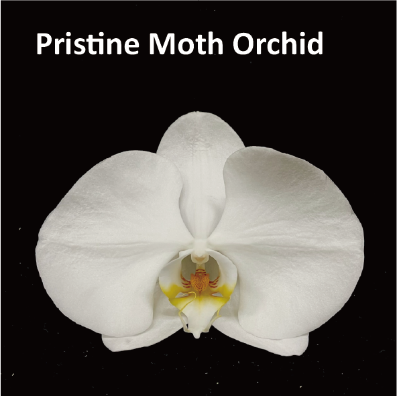
**

**Figure S7.** Initial white moth orchid.


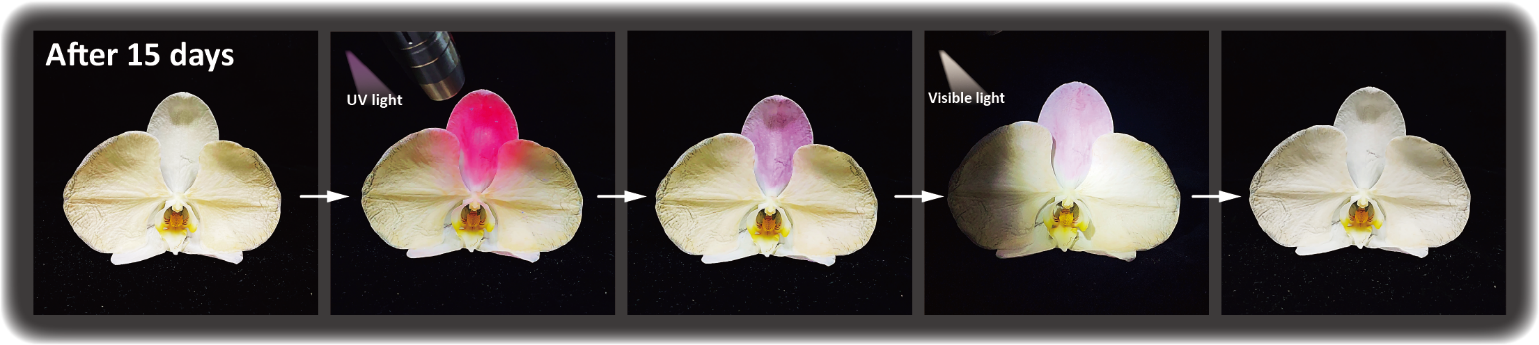


**Figure S8.** Photochromic orchid after storage for 15 days.


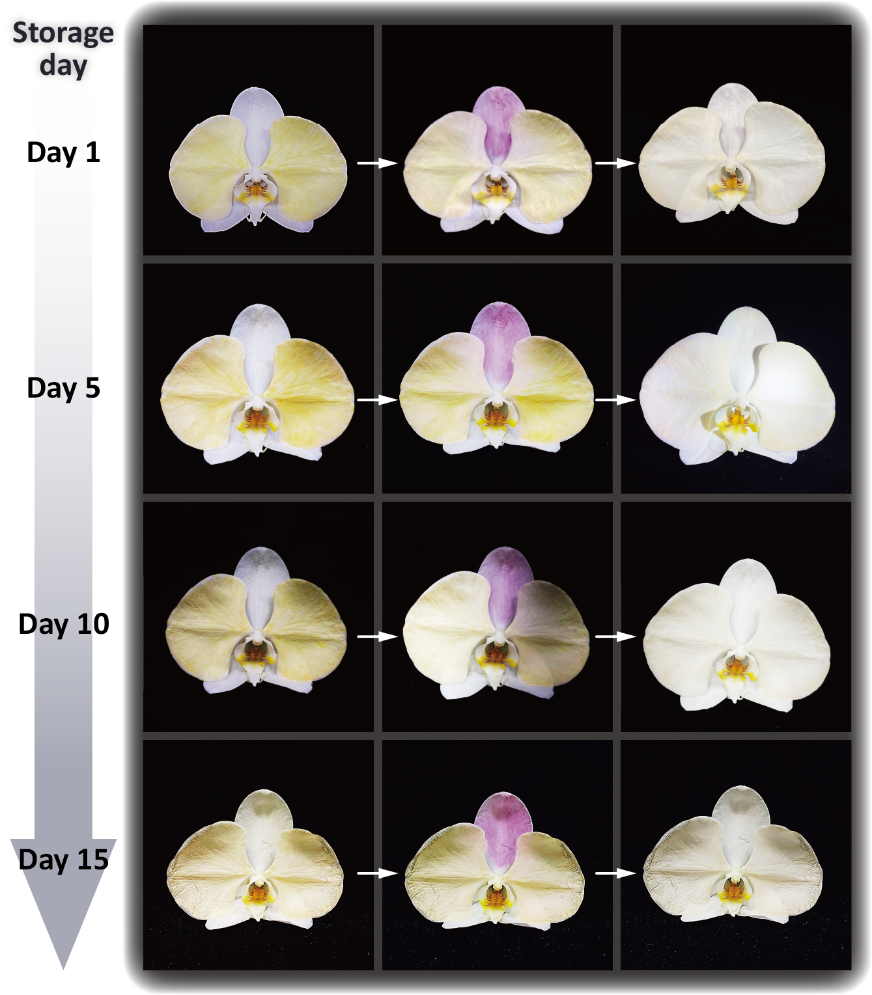


**Figure S9.** Photochromic orchid after different storage times.

**Table S1.** Elemental compositions of a pure TPEE film and thickener, MC-SO_3_, and SP-COOH coated on a TPEE film from XPS analyses

| Samples | Atomic % | | | |
| --- | --- | --- | --- | --- |
|  | C | N | O | S |
| TPEE | 76.22 | 0.09 | 23.63 | 0.05 |
| TPEE thickener | 82.01 | 0.16 | 17.72 | 0.11 |
| TPEE+MC-SO_3_ | 84.74 | 0.71 | 14.00 | 0.55 |
| TPEE+SP-COOH | 81.25 | 0.43 | 17.44 | 0.29 |

The cost of the upcycled TPEE is calculated based on the total cost of its raw materials and processing. The estimated cost of recycled PET, considering the recoverable alcohol used in depolymerization, is approximately $3.35/kg. The cost of petrochemical-based raw materials for conventional TPEE production is about $3.81/kg. The processing costs for both methods, including reaction temperature and time, are similar, adding approximately $0.30/kg. Therefore, the total cost of upcycled TPEE is around $3.65/kg, while the cost of conventional TPEE is approximately $4.11/kg.

**Table S2.** Comparison of the cost of upcycled and conventional TPEE

| Process | Recycled TPEE (this work) | Conventional TPEE |
| --- | --- | --- |
|  | Price (per kg) | |
| Raw material cost | $3.35 | $3.81 |
| Processing cost | $0.30 | $0.30 |
| Estimated total cost | $3.65 | $4.11 |

**
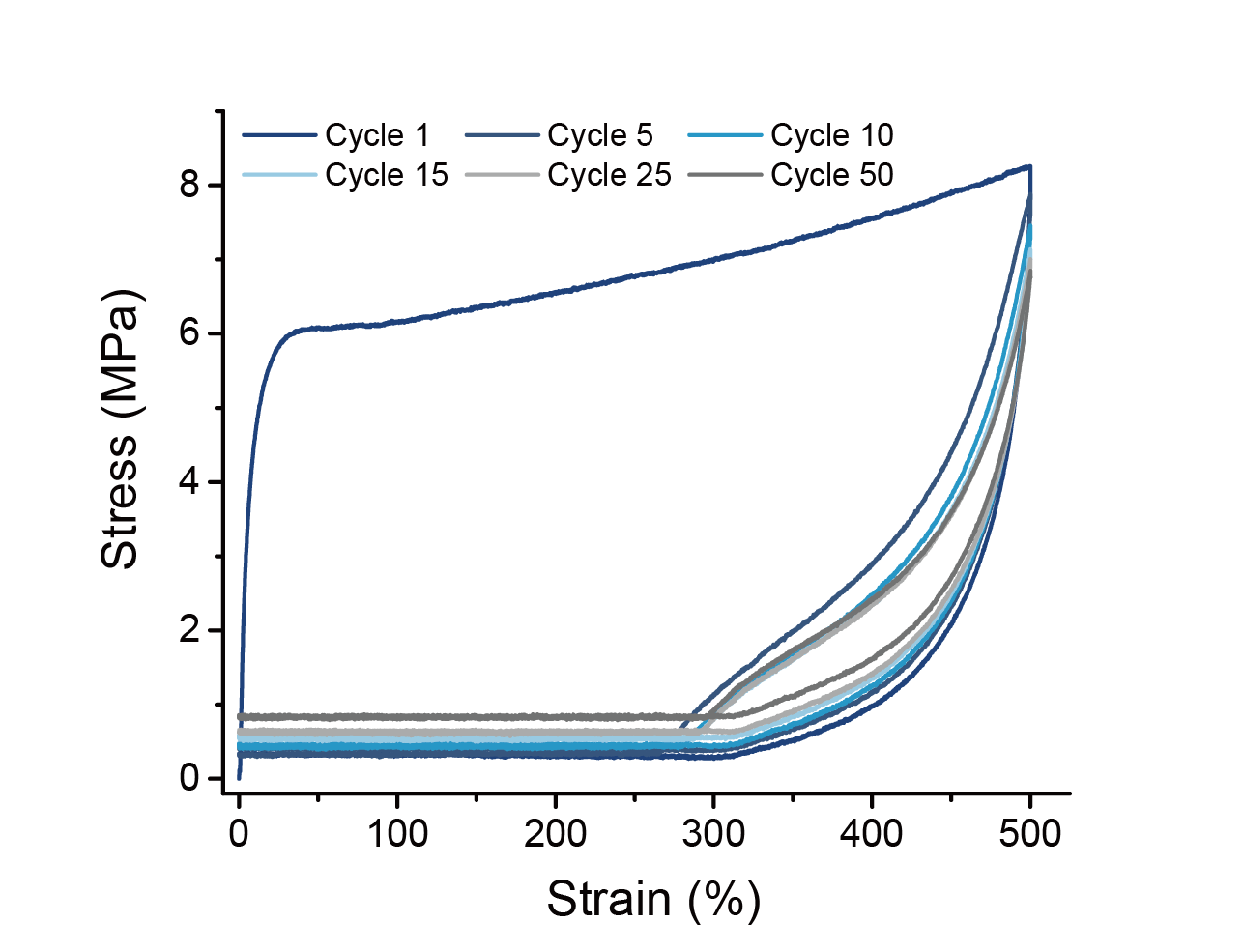
**

**Figure S10.** Stress-strain curves under different cycles of coated TPEE with 500% strain.


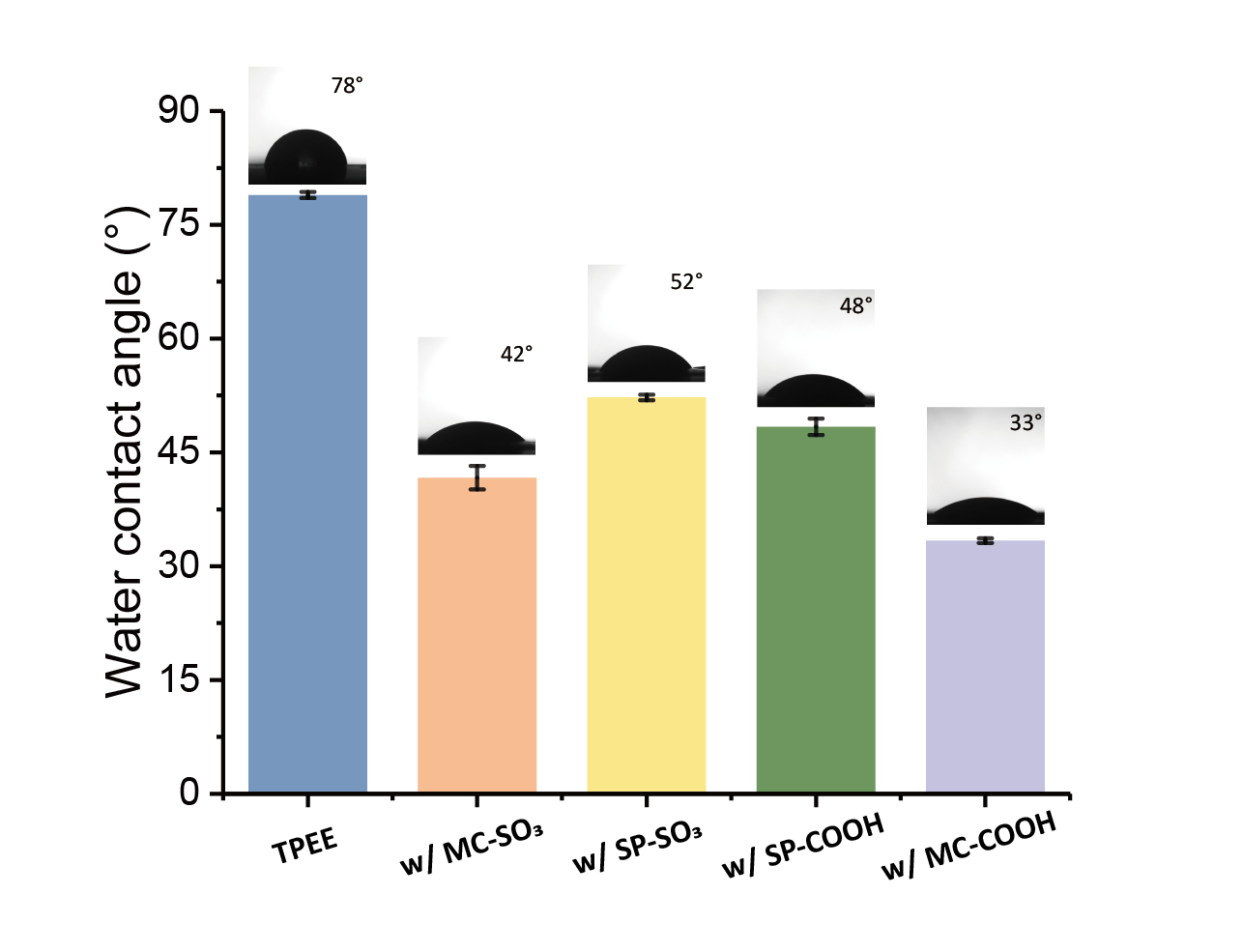


**Figure S11.** Water contact angles of the upcycled TPEE and SP ink-coated TPEE films.

**
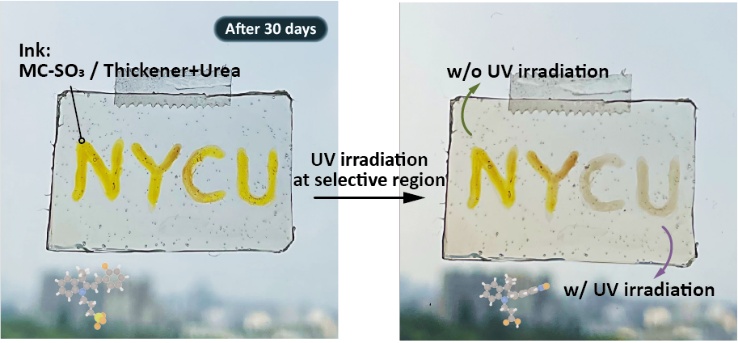
**

**Figure S12.** MC-SO_3_ ink-coated TPEE film with an “NYCU” pattern before and after UV irradiations at selective regions after storage for 30 days.

**
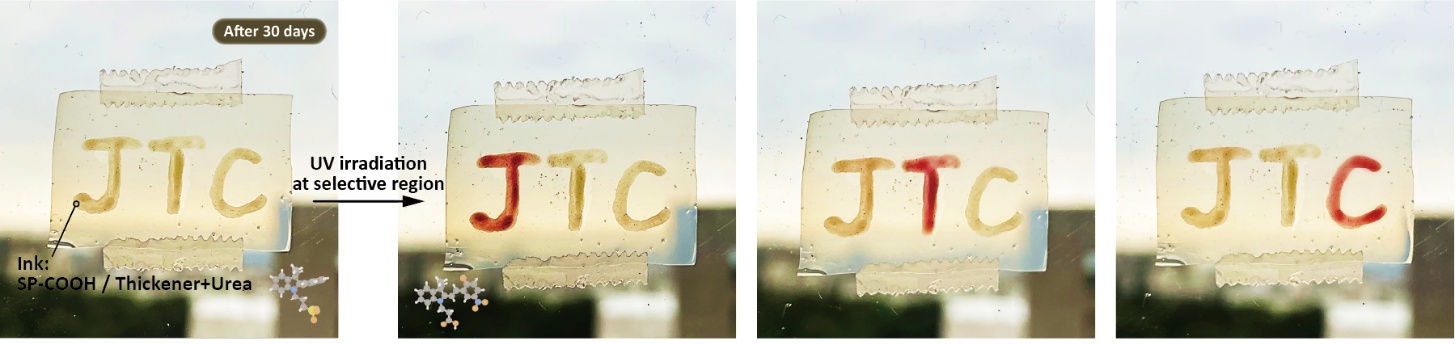
**

**Figure S13.** SP-COOH ink-coated TPEE film with a “JTC” pattern before and after UV irradiations at selective regions after storage for 30 days.


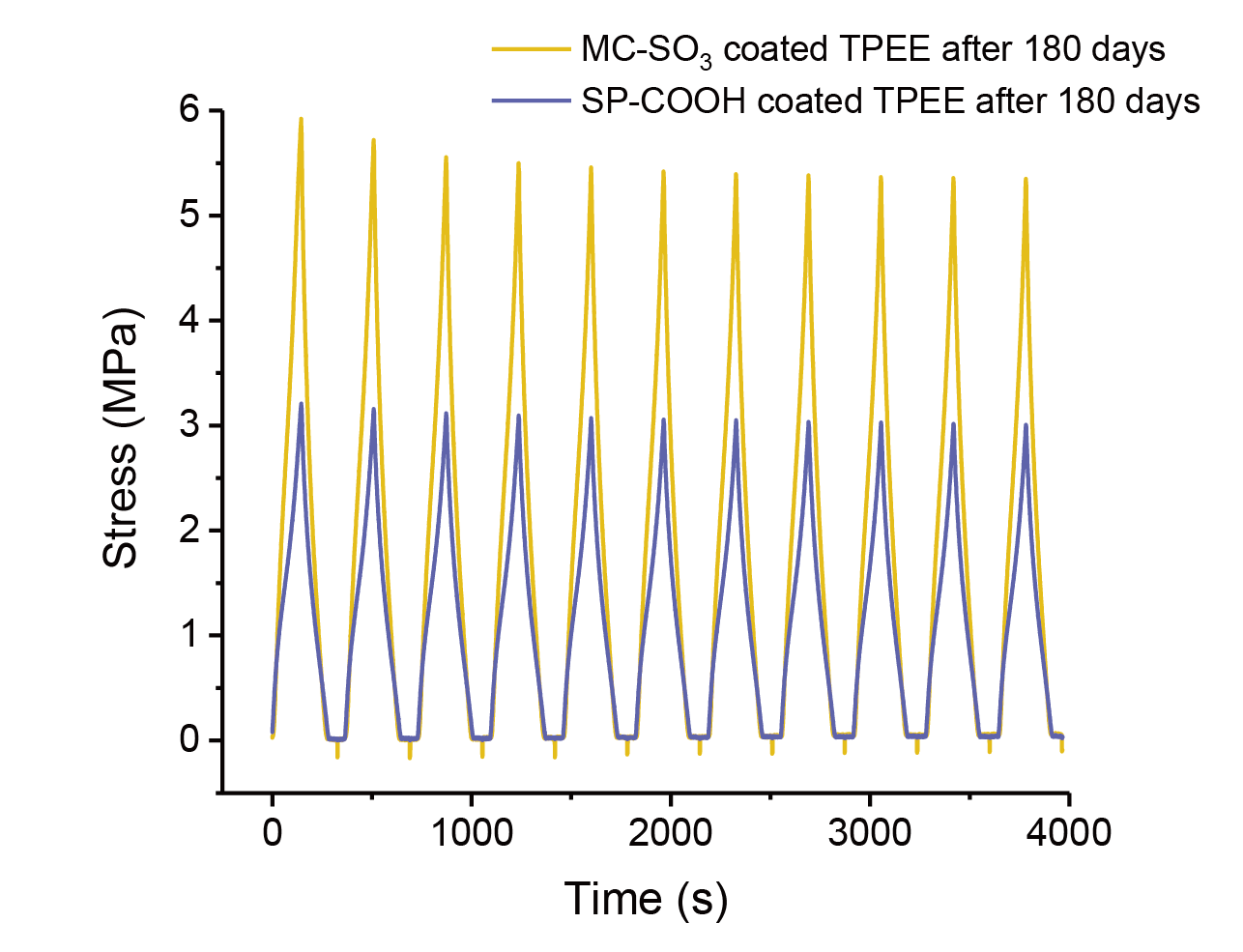


**Figure S14.** Stresses during cyclic tests of the MC-SO_3_ and SP-COOH coated TPEE films after storage for 180 days.

**References**

1. Shi, Z., Peng, P., Strohecker, D. and Liao, Y., Long-Lived Photoacid Based upon a Photochromic Reaction. *J. Am. Chem. Soc.* **2011,** *133*, 14699-14703.

2. Lee, L.-R., Karapala, V. K., Lin, Y.-L., He, H.-C. and Chen, J.-T., Intelligent Environmental Sensing: Fabrication of Switchable, Reusable, and Highly Sensitive Gas Sensors with Spiropyran-Grafted Anodic Aluminum Oxide Templates. *J. Phys. Chem. C* **2020,** *124*, 11870-11876.

3. Guan, X., Yan, S., Chang, J., Yang, G. and Fan, H., Light-induced desorption of trivalent chromium from adsorbents: one step closer to sustainability. *Chem. Commun.* **2018,** *54*, 12770-12773.
